# Research landscape of robot-assisted reconstructive and plastic surgery

**DOI:** 10.1038/s41746-026-03004-7

**Published:** 2026-07-18

**Authors:** Anas A. Sayegh

**Affiliations:** Department of Surgery, College of Medicine, Jazan University, Jazan, Saudi Arabia

**Keywords:** Mathematics and computing, Medical research

## Abstract

Robot-assisted technologies have progressively transformed plastic and reconstructive surgery by enabling enhanced precision, minimally invasive access, and complex microsurgical reconstruction. Despite increasing clinical adoption, a comprehensive data-driven synthesis of global research focused exclusively on original studies remains limited. This study systematically mapped the intellectual structure, collaboration networks, and thematic evolution of robot-assisted plastic and reconstructive surgery using advanced bibliometric approaches. A total of 484 original research articles published between 1996 and 2025 were retrieved from the Scopus database using an optimized search strategy, while secondary document types were excluded to ensure analytical consistency. Descriptive, network, and thematic analyses were performed using Bibliometrix for performance indicators and conceptual mapping, VOSviewer for collaboration and keyword networks, and CiteSpace for co-citation clustering and timeline visualization. The field demonstrated strong growth (annual growth rate 15.89%), with a mean citation impact of 21.6 citations per article and variable field-weighted citation performance across leading authors and institutions. Research output involved 2077 authors across 210 sources, characterized by high collaboration intensity and increasing international participation. The United States dominated productivity and citation impact, followed by China and several European countries. Thematic analyses identified robotic microsurgery as a central research focus, alongside expanding applications in breast reconstruction and transoral robotic surgery, with emerging integration of artificial intelligence.

## Introduction

After a decade of slow but steady adoption^1^, robot-assisted surgery (RAS) now has an expanding role in plastic and reconstructive procedures, with applications spanning complex microsurgery (e.g., lymphatic and nerve repairs), breast reconstruction (robotic mastectomy and flap harvesting), and craniofacial surgery^2–5^. Modern surgical robots provide enhanced three-dimensional visualization, motion scaling, and tremor elimination, enabling precision beyond human limitations while improving surgeon ergonomics through reduced physical strain during lengthy operations^2,6,7^. Recent clinical studies report that robot-assisted techniques can achieve high surgical accuracy and flap survival rates while improving esthetic outcomes and patient satisfaction^8^, often with complication rates equal to or lower than conventional approaches^6^. These advances have enabled minimally invasive approaches that preserve critical anatomical structures and reduce donor-site morbidity without compromising reconstructive outcomes^1,9^. However, significant challenges persist, including steep learning curves, prolonged operative times, high equipment costs, and the absence of tactile feedback, which currently limit widespread adoption^10^. Ongoing technological refinements—such as more compact, cost-effective systems and integration of artificial intelligence and haptic feedback—combined with further high-quality clinical studies (including prospective trials) are needed to overcome these barriers and fully establish the clinical value of robotics in plastic and reconstructive surgery (RAPRS)^11–13^.

Despite the growing interest in intelligent and robot-assisted technologies within RAPRS, the existing bibliometric literature reveals important gaps that limit a comprehensive understanding of this evolving field. Previous studies have either adopted a broad artificial intelligence–centered perspective, in which robotic surgery represents only one component among multiple digital innovations^14^, or have focused narrowly on specific disease domains such as robotic surgery in breast cancer, thereby overlooking the wider spectrum of reconstructive, microsurgical, and esthetic applications relevant to plastic surgery as a specialty^15^. Although a recent bibliometric analysis has explicitly examined robot-assisted surgery in plastic surgery, it was constrained by a relatively small dataset, reliance on a single database and analytical platform, and inclusion of heterogeneous document types, which may dilute insights derived from primary empirical evidence^16^.

Consequently, there remains a clear need for a methodologically rigorous, data-driven scientometric study that systematically maps the intellectual and structural landscape of robot-assisted plastic and reconstructive surgery research. Accordingly, the primary objective of this study is to conduct a comprehensive scientometric mapping of RAPRS literature based exclusively on original research articles. Using an optimized Scopus-based search strategy and integrated bibliometric platforms (Bibliometrix, VOSviewer, and CiteSpace), the study examines publication productivity, collaboration networks, citation structure, and thematic evolution to characterize the field’s knowledge architecture and research dynamics. This approach aims to generate a high-resolution overview of global research trends, influential contributors, and emerging research directions in RAPRS.

This investigation adopts an inclusive, procedure-centered rather than specialty-centered definition of RAPRS. This procedure-centered definition was adopted to capture robotic microsurgical innovations that are technically transferable to reconstructive surgery, particularly in areas such as supermicrosurgical anastomosis, flap-based reconstruction, and minimally invasive tissue access, where methodological advances frequently originate from adjacent specialties, including otolaryngology and urology. The analysis encompasses robotic applications relevant to reconstructive surgical principles—including tissue transfer, microsurgical anastomosis, and esthetic restoration—regardless of the specialty discipline of origin. Consequently, transoral robotic surgery (otolaryngology), robotic microsurgical anastomosis (urology), and robotic-assisted free flap procedures (multiple specialties) are included based on shared technical foundations and transferable microsurgical paradigms applicable to plastic surgical reconstruction. This approach acknowledges the inherently multidisciplinary nature of surgical robotics innovation, in which technological advances in adjacent specialties inform the evolution of reconstructive practice.

## Results

### Descriptive analysis

The bibliometric analysis comprised 484 original research articles published between 1996 and 2025, drawn from 210 sources, including peer-reviewed journals and related outlets. The field demonstrated a strong and sustained expansion, with an annual growth rate of 15.9%, indicating rapidly increasing scholarly interest in robot-assisted plastic and reconstructive surgery (RAPRS). The average document age was 5.84 years, reflecting the contemporary nature of the research, while the mean citation count per document reached 21.6, suggesting moderate to high scientific impact. A total of 2077 authors contributed to the literature, with only 11 single-authored articles, highlighting the field's collaborative nature. The mean number of co-authors per document was 6.2, and international collaborations accounted for 20.45% of publications, underscoring growing global research integration.

### Annual publication and citation trends

**Fig. 1:**
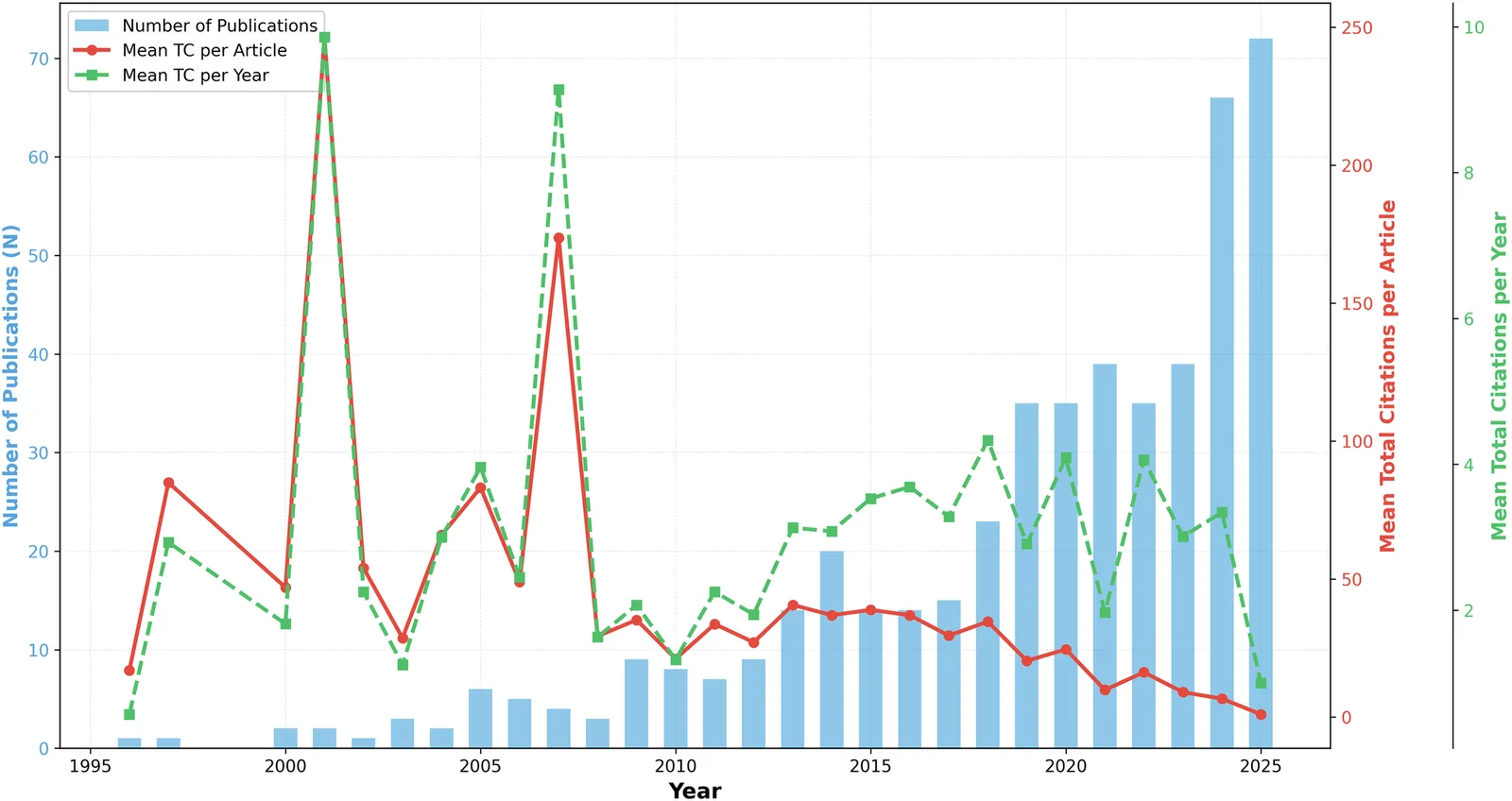
Temporal trends in publication output and citation metrics (1996–2025). The primary *y*-axis depicts annual publication volume (blue bars), the secondary *y*-axis represents mean total citations per article (red line), and the tertiary *y*-axis illustrates mean total citations per year (green dashed line).

Publications were distributed across a 30-year observation period (1996–2025), yielding a mean annual output of 17.29 articles (Fig. 1). Publication productivity demonstrated substantial temporal variation, with a peak of 72 articles recorded in 2025. As data for 2025 represent partial-year coverage (January–November), this value should be interpreted as reflecting recent publication momentum rather than a direct comparison with complete calendar years. Citation metrics also varied considerably over time. Mean total citations per article reached a maximum of 246.50 in 2001, while mean total citations per year peaked at 9.86 during the same period. These elevated citation values were primarily driven by a small number of highly influential early publications and the longer citation accumulation period available to articles published in the early years of the field. In contrast, more recent publications have had less time to accrue citations. Therefore, differences in citation performance across publication years should be interpreted mainly as citation-window effects rather than evidence of greater scientific impact or research productivity in earlier periods. Overall, the temporal distribution demonstrates accelerating publication growth in recent years, whereas citation influence remains concentrated among foundational studies published during the early development of RAPRS research.

### Global research productivity, co-authorship, and citation

**Fig. 2:**
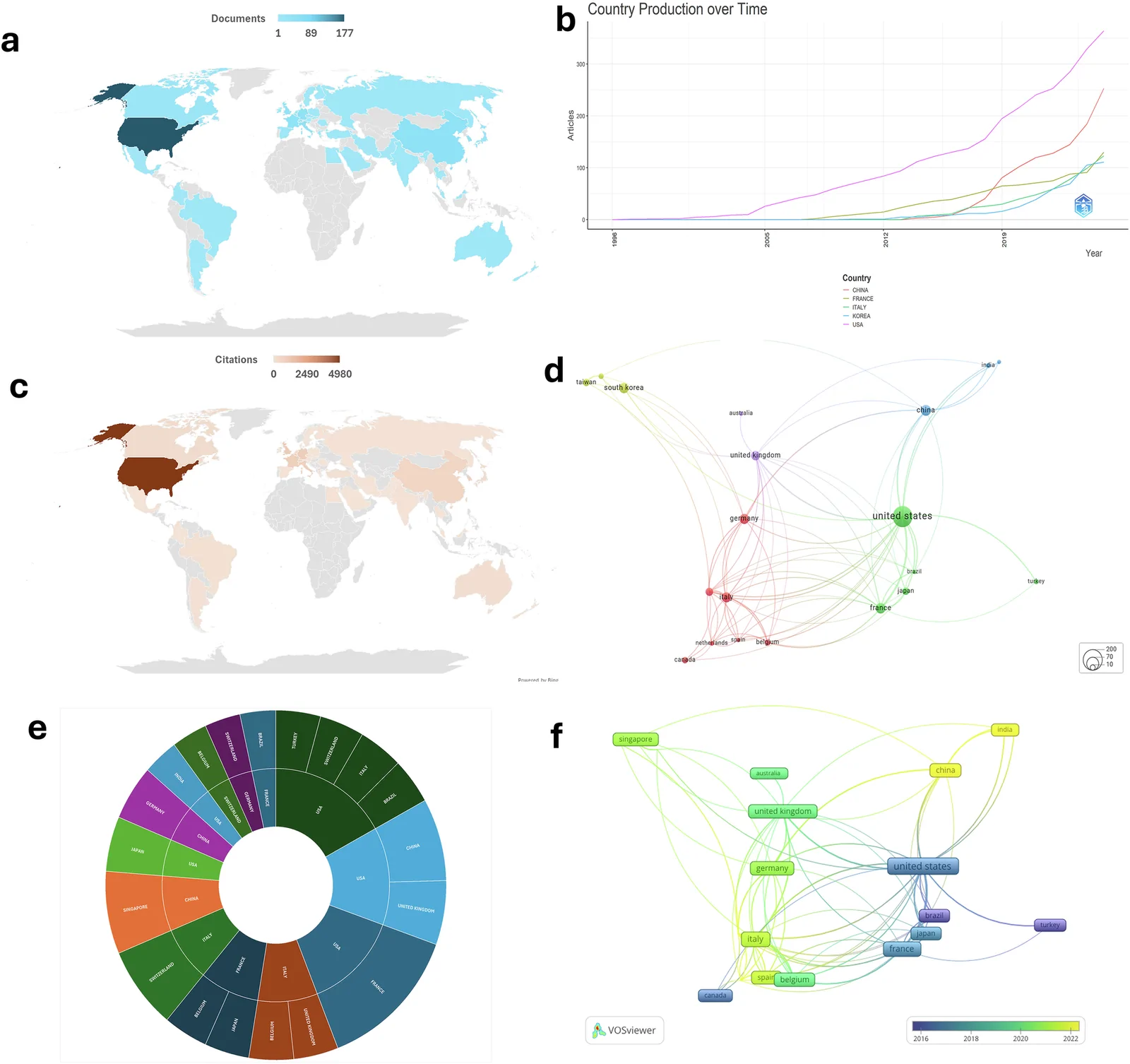
Global distribution of research productivity, citation impact, and international collaboration networks in robot-assisted plastic and reconstructive surgery. **a** depicts geographic distribution of publication output (*N* = 484 documents). **b** illustrates temporal trajectories of the five most prolific nations (1996–2025), demonstrating exponential growth patterns with USA dominance. **c** presents the global citation distribution (13,276 total citations), revealing North American and European concentration. **d** visualizes international collaboration networks generated through VOSviewer, displaying 77 linkages across 20 countries organized into five distinct clusters. An interactive version of this figure is available at https://tinyurl.com/2cm7xx8z. **e** represents bilateral collaboration frequencies among contributing nations. **f** demonstrates the temporal evolution of collaborative engagement (2016–2022), with color gradation indicating chronological progression from established (blue) to emerging (yellow) partnerships.

Global research productivity in RAPRS was distributed across 45 countries and six continents (Fig. 2a). Europe contributed the largest share of publications (*n* = 243, 38.1%), followed by North America (*n* = 194, 30.4%) and Asia (*n* = 177, 27.7%). The United States was the leading contributor (*n* = 177, 27.7%), followed by China (*n* = 46, 7.2%), France (*n* = 44, 6.9%), South Korea (*n* = 42, 6.6%), and Germany and Italy (*n* = 40 each, 6.3%).

Figure 2b illustrates the temporal trajectories of the leading contributing countries. The United States demonstrated sustained growth throughout the study period, whereas China showed accelerated expansion after 2015. France, Italy, and South Korea exhibited steady increases in cumulative publication output, reaching 130, 123, and 111 publications, respectively, by 2025.

Global citation impact totaled 13,276 citations across 45 countries (Fig. 2c). North America accounted for the largest share of citations (*n* = 5277, 39.8%), followed by Europe (*n* = 5035, 37.9%) and Asia (*n* = 2557, 19.3%). The United States accumulated the highest citation count (*n* = 4980, 37.5%), followed by the United Kingdom (*n* = 948, 7.1%), Germany (*n* = 838, 6.3%), France (*n* = 805, 6.1%), and Italy (*n* = 749, 5.6%). South Korea was the highest-cited Asian country (*n* = 705, 5.3%). South America, Oceania, and Africa collectively accounted for less than 3% of total citations, highlighting substantial geographic disparities in research impact.

International collaboration network analysis identified 20 countries exceeding the minimum threshold of five publications, forming 77 collaborative linkages with a cumulative total link strength (TLS) of 438 distributed across five clusters (Fig. 2d). The United States demonstrated the highest collaborative engagement (TLS = 77), followed by France (TLS = 43), Italy (TLS = 40), and Switzerland (TLS = 38). European countries formed densely interconnected collaboration networks, whereas Asian countries generally occupied more peripheral positions with lower linkage strengths.

Bilateral collaboration analysis identified 137 international partnerships spanning multiple continents (Fig. 2e). The strongest collaboration was observed between the United States and France (*n* = 16 co-authored publications), followed by China–Singapore, Italy–Switzerland, and United States–China partnerships (*n* = 9 each). European countries exhibited dense intra-regional collaborations, whereas transcontinental partnerships were predominantly centered on the United States.

The overlay visualization (Fig. 2f) depicts the temporal evolution of international collaborations between 2016 and 2022. More recent collaborative activity was concentrated among Asian contributors, particularly China, India, and Singapore, whereas collaborations involving the United States, United Kingdom, and Germany were established earlier, indicating a gradual expansion of the global RAPRS collaboration network.

### Prolific affiliations

Institutional productivity analysis revealed the ten most prolific affiliations contributing to RAPRS research. Yonsei University College of Medicine (South Korea, *n* = 16) and Universitätsspital Zürich (Switzerland, *n* = 16) demonstrated the highest publication output, followed by Les Hôpitaux Universitaires de Strasbourg (France, *n* = 15) and The University of Texas MD Anderson Cancer Center (USA, *n* = 14). Johns Hopkins University (USA, *n* = 12), University of Pennsylvania (USA, *n* = 11), and Cleveland Clinic Foundation (USA, *n* = 11) occupied subsequent positions. Four institutions contributed nine publications each: Johns Hopkins University School of Medicine (USA), Chang Gung University (Taiwan), University of Texas Health Science Center at Houston (USA), and Chang Gung Memorial Hospital (Taiwan). USA institutions dominated the ranking with six of the ten positions, while Asian and European institutions demonstrated concentrated excellence, collectively representing international leadership in robotic surgical innovation within reconstructive and esthetic surgical domains.

### Author-level analysis: productivity distribution, collaboration networks, and intellectual structure

**Fig. 3:**
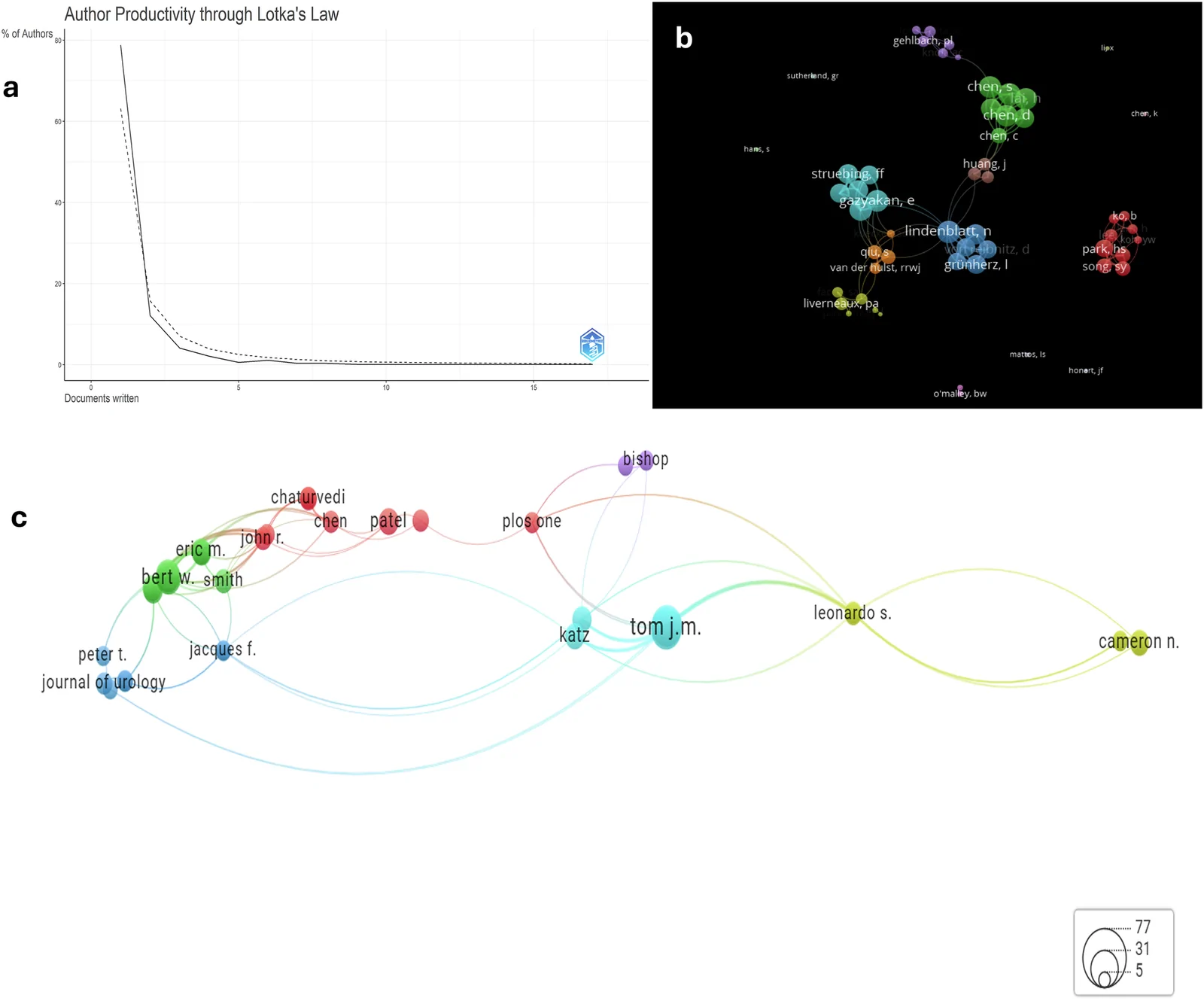
Author productivity distribution and collaboration networks in robot-assisted plastic and reconstructive surgery research. **a** depicts the empirical author productivity distribution compared to Lotka’s theoretical law (dashed line), demonstrating marked deviation with 78.8% single-publication authors. **b** illustrates the VOSviewer-generated author collaboration network (minimum five publications threshold) comprising 55 authors, 120 linkages, and 15 clusters, with node size representing publication volume and link thickness indicating collaboration strength. An interactive version of this figure is available at https://tinyurl.com/2ytssjn3. **c** presents an author co-citation network encompassing 36 highly cited authors across six thematic clusters (122 linkages, total link strength = 543), revealing intellectual structure fragmentation across surgical subspecialties. Node coloration denotes cluster membership; edge proximity indicates co-citation frequency intensity. An interactive version of this figure is available at https://tinyurl.com/2yxurh8g.

Author productivity distribution analysis revealed substantial deviation from Lotka’s theoretical law, which postulates that the proportion of authors producing *n* publications approximates 1/*n*² of single-publication authors (Fig. 3a). The observed empirical distribution demonstrated marked concentration among occasional contributors, with 78.8% of authors (*n* = 1637) contributing single publications, substantially exceeding Lotka’s theoretical prediction of approximately 60%. Authors producing two publications constituted 12.1% (*n* = 252), while three-publication authors represented 4.1% (*n* = 85). This pronounced skewness toward single-publication authorship may reflect several structural characteristics of RAPRS research rather than indicating limited sustained engagement by individual investigators. Possible contributing factors include platform-based publication patterns in which investigators contribute selectively to robotic surgery within broader surgical research portfolios, trainee-driven institutional output with rotating personnel, and multidisciplinary participation across surgical specialties.

The ten most prolific authors contributing to RAPRS research demonstrated substantial heterogeneity in scholarly productivity and citation impact metrics (Table 1). Selber, J.C. (Corewell Health East, USA) emerged as the most productive investigator with 14 domain-specific publications, followed by Lindenblatt, N. (Universitätsspital Zürich, Switzerland, *n* = 12) and Liverneaux, P. (Université de Strasbourg, France, *n* = 11). Park, H.S. (Yonsei University, South Korea) contributed ten publications, whilst Giovanoli, P. (Universitätsspital Zürich, Switzerland) generated nine. Notably, Kneser, U. demonstrated the highest overall scholarly output (605 total publications) and citation accumulation (12,534 citations), despite contributing eight domain-specific articles. Field-Weighted Citation Impact values exhibited considerable variation, ranging from 0.318 to 3.14, with Lindenblatt, N. attaining the maximum FWCI (3.14), indicating citations exceeding threefold the global disciplinary baseline. FWCI values reported as 0 reflect insufficient citation data or database coverage for reliable metric calculation rather than indicating the absence of scholarly impact. Geographic representation encompassed the USA, Switzerland, France, South Korea, Taiwan, and Germany.

**Table 1:** Bibliometric characteristics of the ten most prolific authors in RAPRS research

No	Author	Affiliation	*N*	Total publication	Total citation	FWCI	*H*-Index	Scopus ID
1	Selber, J.C.	Corewell Health East, Royal Oak, USA	14	230	6091	1.489	43	35588200000
2	Lindenblatt, N.	Universitätsspital Zürich, Zürich, Switzerland	12	174	3419	3.14	31	9534382600
3	Liverneaux, P.	iCube, Université de Strasbourg, France	11	308	3250	0.802	29	12768900500
4	Park, H.S.	Yonsei University College of Medicine, Seoul, South Korea	10	170	3648	1.316	32	55713697800
5	Giovanoli, P.	Universitätsspital Zürich, Zürich, Switzerland	9	297	6216	0	43	7003525131
6	Chen, D.R.	Changhua Christian Hospital, Changhua, Taiwan	8	266	7953	0.342	47	55549584700
7	Chen, S.T.	Changhua Christian Hospital, Changhua, Taiwan	8	155	6408	0.364	43	26642967600
8	Gazyakan, E.	Klinikum Ludwigshafen, Ludwigshafen, Germany	8	117	1930	0.345	23	8986499400
9	Kneser, U.	BG-Unfallklinik Ludwigshafen, Ludwigshafen, Germany	8	605	12,534	0.318	56	26643400800
10	Lai, H.W.	Changhua Christian Hospital, Changhua, Taiwan	8	92	2395	2.635	29	8918281200

*N* represents the number of publications specific to robot-assisted plastic and reconstructive surgery. Total Publication denotes cumulative scholarly output across all research domains. *H*-Index quantifies an author’s productivity and citation impact, defined as the maximum value *h* whereby the author has published h papers that have each received at least *h* citations. Field-weighted citation impact (FWCI) normalizes citation performance relative to global disciplinary averages, with values exceeding 1.0 indicating above-average citation impact within the respective field. FWCI = 0 indicates insufficient citation data availability or database coverage for reliable metric calculation, not the absence of scholarly impact. FWCI calculation requires minimum citation thresholds and subject classification matching; values may be unavailable for recently published work or publications in interdisciplinary domains lacking clear subject category assignment. Author affiliations reflect primary institutional associations at the time of data extraction.

Author collaboration network analysis employing VOSviewer methodology with a minimum threshold of five publications identified 55 authors from the total corpus of 2027 investigators (Fig. 3b). The network comprised 55 nodes, 120 collaborative linkages, and a cumulative total link strength of 466, distributed across 15 distinct clusters. The largest connected component encompassed 38 authors, indicating a fragmented collaboration architecture with 17 authors occupying peripheral or isolated positions. Prominent collaborative nuclei centered around Lindenblatt, *N* (total link strength = 42), Chen, *D* (TLS = 40), Chen, *S* (TLS = 40), and Lai, *H* (TLS = 39). Additional densely interconnected clusters emerged around Gazyakan, E (TLS = 38), Kneser, *U* (TLS = 38), Struebing, FF (TLS = 33), Park, HS, and Liverneaux, PA, demonstrating geographically concentrated research consortia predominantly representing European and Asian institutional networks.

Author co-citation network analysis utilizing VOSviewer methodology identified 36 highly cited authors distributed across six thematic clusters, interconnected through 122 co-citation linkages with a cumulative total link strength of 543 (Fig. 3c). Cluster composition ranged from four to eight authors, with Cluster 1 encompassing eight seminal contributors, including foundational journal sources (Frontiers in Oncology, PLOS ONE). Tom, J.M. and Van Mulken emerged as the most extensively co-cited authors (38 citations each), followed by Bert, W. and O’Malley (24 citations each). Cluster 2 comprised seven authors representing transoral robotic surgery scholarship (O’Malley, Hockstein, Neil, G., Genden). Cluster 3 incorporated urological robotics literature (Journal of Urology, Marescaux, Ballantyne), while Clusters 4-6 represented specialized microsurgical and reconstructive surgical domains, collectively demonstrating intellectual structure fragmentation across multiple surgical subspecialties and methodological approaches within RAPRS research.

### Analysis of sources

**Fig. 4:**
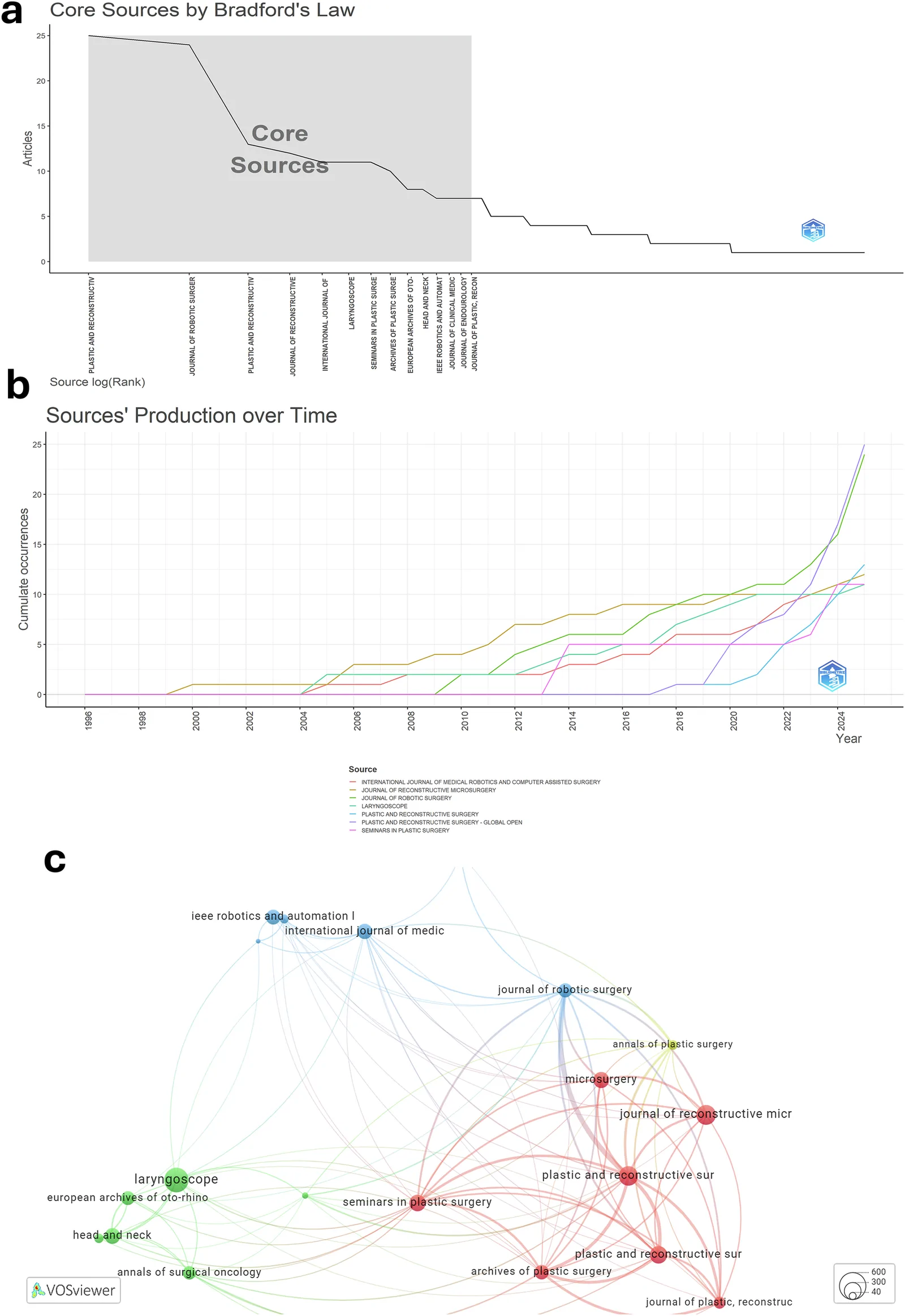
Source distribution, temporal dynamics, and co-citation network analysis in robot-assisted plastic and reconstructive surgery research. **a** illustrates Bradford’s Law source stratification, delineating three hierarchical zones with a shaded region indicating 14 core journals contributing 161 publications (33.3% of corpus). **b** depicts cumulative publication trajectories for the seven most productive sources spanning 1996–2025, demonstrating exponential growth post-2018. **c** presents VOSviewer-generated source co-citation network comprising 20 journals across four thematic clusters (103 linkages, total link strength = 938), with node size representing citation frequency and cluster coloration denoting disciplinary subspecialty groupings. Network architecture reveals intellectual structure fragmentation across plastic surgery, otolaryngology, and robotics engineering domains. An interactive version of this figure is available at https://tinyurl.com/2cjsgfjm.

Bradford’s Law source distribution analysis delineated three hierarchical zones encompassing 210 journals contributing to RAPRS literature (Fig. 4a). Zone 1 comprised 14 core journals (ranks 1–14) generating 161 publications (33.3% of corpus), led by Plastic and Reconstructive Surgery—Global Open (*n* = 25), Journal of Robotic Surgery (*n* = 24), and Plastic and Reconstructive Surgery (*n* = 13). Zone 2 encompassed 53 journals (ranks 15–67) contributing 166 publications, while Zone 3 contained 144 peripheral journals (ranks 68–211) yielding 157 publications. The ten most impactful sources demonstrated substantial bibliometric heterogeneity (Table 2), with h-indices ranging from 6 to 10. Journal of Reconstructive Microsurgery, Laryngoscope, and Plastic and Reconstructive Surgery—Global Open achieved the highest h-index values (*h* = 10). Temporal source dynamics revealed exponential growth trajectories post-2018 across core journals, with cumulative publications reaching 25 by 2025 for leading sources (Fig. 4b).

**Table 2:** Source impact

Source	H_index	G_index	M_index	Tc	Np	Py_start
*Journal of Reconstructive Microsurgery*	10	12	0.385	348	12	2000
*Laryngoscope*	10	11	0.476	538	11	2005
*Plastic and Reconstructive Surgery—Global Open*	10	18	1.25	338	25	2018
*Journal of Robotic Surgery*	8	12	0.5	177	24	2010
*Archives of Plastic Surgery*	7	10	0.538	186	10	2013
*Plastic and Reconstructive Surgery*	7	13	0.875	274	13	2018
*European Archives of Oto-Rhino-Laryngology*	6	8	0.462	170	8	2013
*IEEE Robotics and Automation Letters*	6	7	0.75	195	7	2018
*International Journal of Medical Robotics and Computer-Assisted Surgery*	6	11	0.286	206	11	2005
*Journal of Plastic, Reconstructive and Esthetic Surgery*	6	7	1	128	7	2020

H_index: Hirsch index indicating the number of publications (Np) that have received at least the same number of citations; G_index: Egghe’s index reflecting the global citation performance of the most cited publications; M_index: H_index normalized by the number of years since the first publication; Tc: total number of citations received; Np: number of publications; Py_start: first year of publication within the dataset.

Source co-citation network analysis employing VOSviewer methodology with a minimum threshold of five publications identified 20 journals from the corpus of 210 sources (Fig. 4c). The network comprised 20 nodes interconnected through 103 co-citation linkages with a cumulative total link strength of 938, distributed across four thematic clusters. Cluster 1 encompassed seven plastic and reconstructive surgery journals, including Journal of Reconstructive Microsurgery, Plastic and Reconstructive Surgery, and Microsurgery, representing the disciplinary core. Cluster 2 comprised six head and neck oncology journals (Laryngoscope, Head and Neck, European Archives of Oto-Rhino-Laryngology). Cluster 3 incorporated six robotics and engineering journals (IEEE Robotics and Automation Letters, Journal of Robotic Surgery), while Cluster 4 contained Annals of Plastic Surgery, demonstrating intellectual structure stratification across surgical subspecialties and technological domains.

### Knowledge organization and conceptual relationships: index keyword co-occurrence patterns

**Fig. 5:**
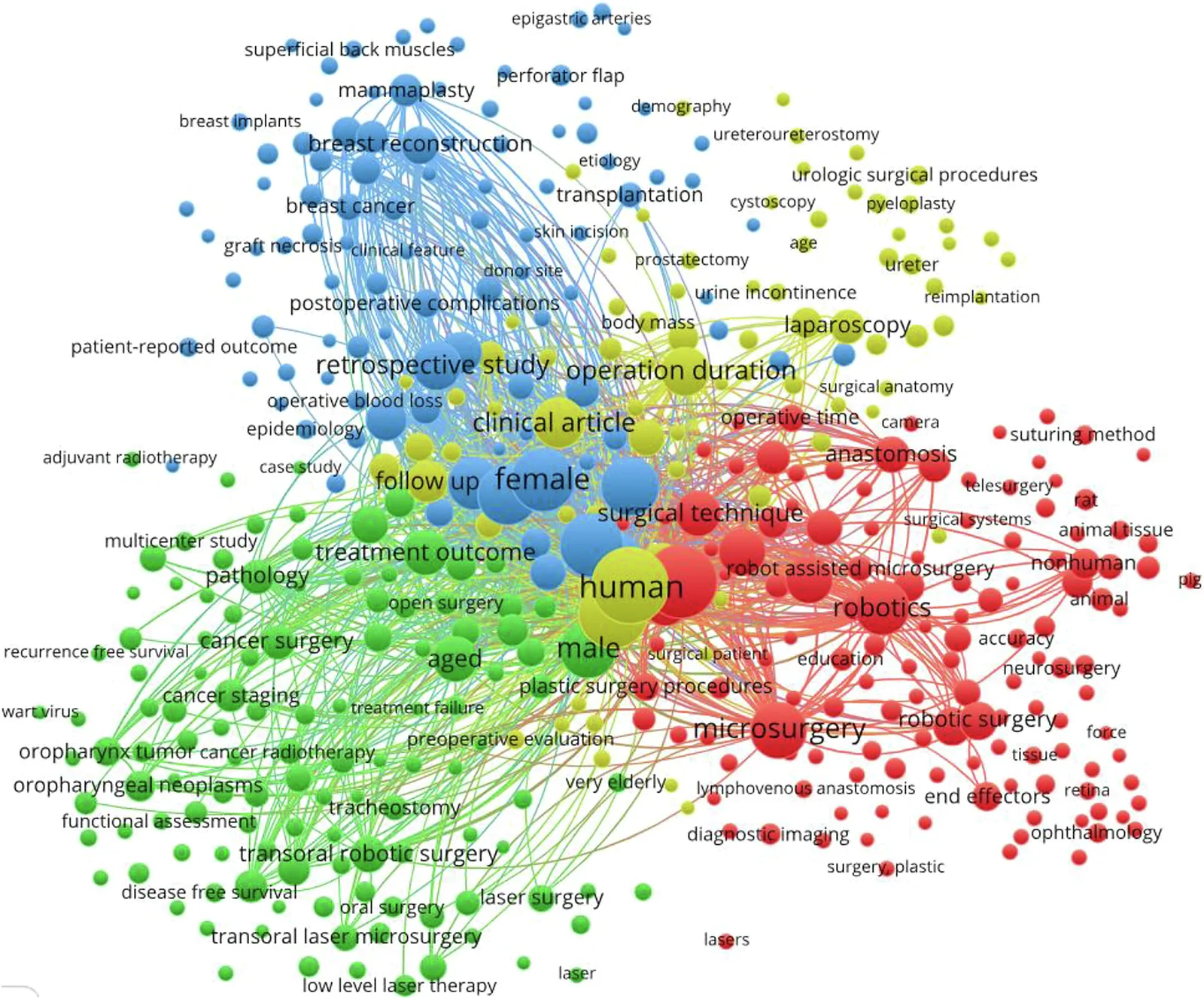
Index keyword co-occurrence network visualization depicting the conceptual structure of robot-assisted plastic and reconstructive surgery research. VOSviewer-generated network comprises algorithmically assigned database index keywords, revealing four thematic clusters distinguished by chromatic differentiation: red (robotic microsurgical techniques and technical instrumentation), blue (breast reconstruction and oncoplastic surgery), green (transoral robotic surgery and head-neck oncology), yellow (urological robotic procedures and laparoscopic applications). Node size reflects keyword occurrence frequency; edge thickness indicates co-occurrence strength. Central positioning of terms including “human,” “surgical technique,” and “follow up” demonstrates cross-cluster bridge concepts integrating diverse subspecialty domains. Network architecture reveals both disciplinary fragmentation across specialized surgical applications and conceptual integration through shared methodological paradigms.

Index keyword co-occurrence network analysis employing VOSviewer methodology with a minimum threshold of five occurrences identified 430 keywords from the total corpus of 3261 database-assigned terms (Fig. 5). The network comprised 430 nodes interconnected through 31,517 co-occurrence linkages with a cumulative total link strength of 117,563, distributed across four principal thematic clusters. The red cluster, positioned in the right hemisphere, encompassed robotic microsurgical techniques anchored by “microsurgery,” “robotic surgery,” “robot-assisted microsurgery,” and “anastomosis,” incorporating technical instrumentation terminology including “surgical systems,” “telesurgery,” “accuracy,” and “end effectors.” The blue cluster concentrated on breast reconstruction and oncoplastic surgery, dominated by “breast reconstruction,” “breast cancer,” “mammaplasty,” and “postoperative complications,” revealing strong conceptual associations between oncological diagnosis and reconstructive interventions. The green cluster represented transoral robotic surgery and head-neck oncology, incorporating “transoral robotic surgery,” “oropharyngeal neoplasms,” “cancer staging,” and “treatment outcome,” emphasizing oncological efficacy metrics. The yellow cluster delineated urological robotic procedures, centered on “laparoscopy,” “urologic surgical procedures,” and “pyeloplasty.” High-centrality nodes, including “human,” “male,” “female,” “surgical technique,” and “follow up,” demonstrated substantial cross-cluster connectivity, indicating shared demographic parameters and methodological frameworks transcending subspecialty boundaries, thereby revealing both thematic heterogeneity and conceptual integration within RAPRS scholarship.

### Temporal dynamics analysis

**Fig. 6:**
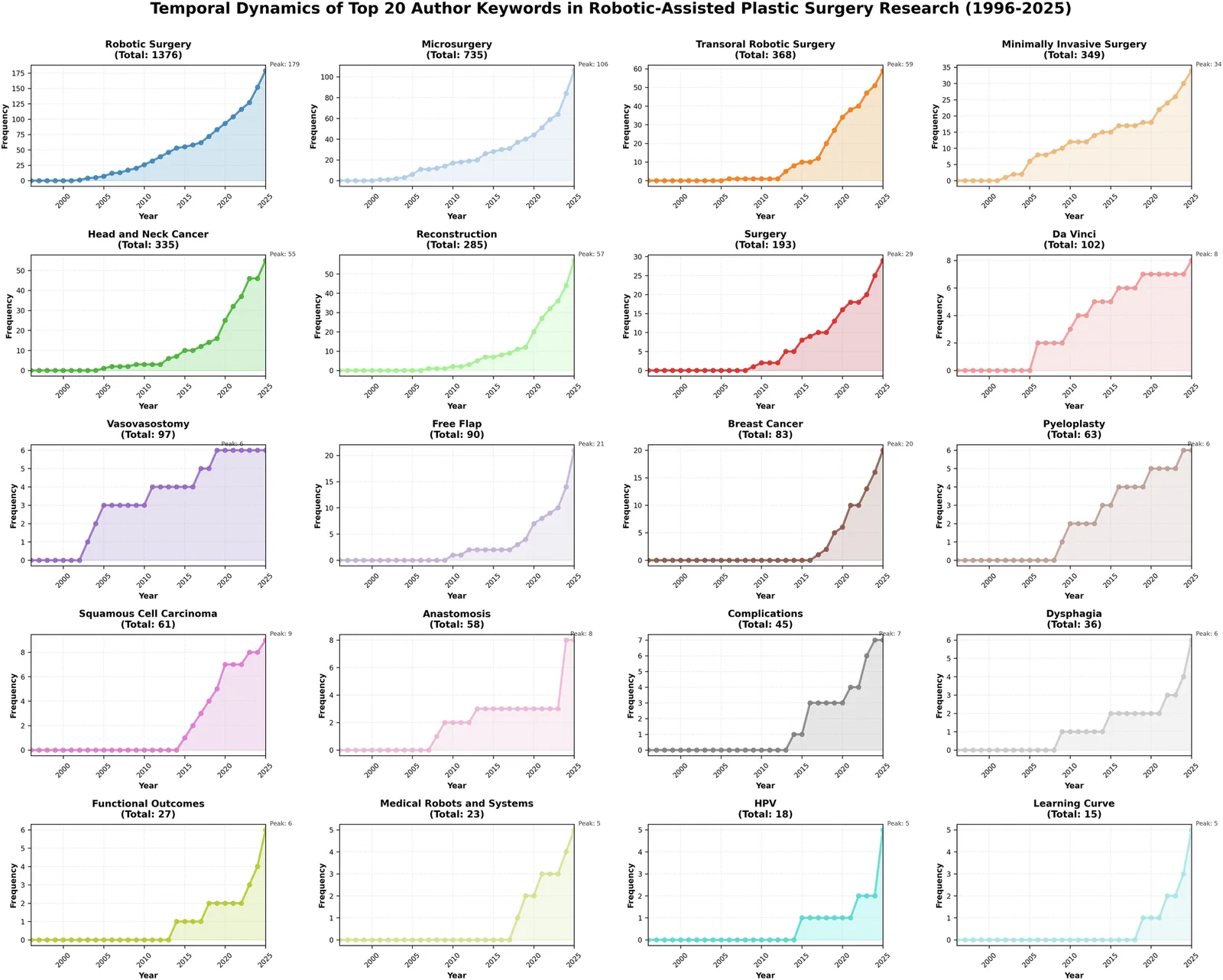
Temporal dynamics of the top 20 consolidated author keywords in robot-assisted plastic and reconstructive surgery research (1996–2025). Individual panels display annual occurrence frequencies for each keyword, with area shading indicating cumulative trends. Keywords represent consolidated terms merging semantically related variants (e.g., robotic surgery encompasses eight distinct terminology iterations). Peak frequency values are annotated within respective temporal positions. Total cumulative occurrences appear in panel titles. Seventeen keywords achieved maximum frequencies during 2024–2025, demonstrating concentrated recent research intensification across dominant thematic domains, including robotic surgery, microsurgery, and transoral robotic surgery applications.

Temporal dynamics analysis of consolidated author keywords encompassing the top 20 most frequently occurring terms revealed pronounced evolutionary trajectories across the 30-year observation period (Fig. 6). Robotic Surgery demonstrated the highest cumulative frequency (*n* = 1376 occurrences), with peak annual occurrence of 179 in 2025, followed by Microsurgery (*n* = 735, peak=106 in 2025) and Transoral Robotic Surgery (*n* = 368, peak = 59 in 2025). Keyword entry points exhibited temporal stratification, with foundational terms including Microsurgery (2001) and Robotic Surgery (2002) establishing early presence, while contemporary themes such as Breast Cancer (2017), Medical Robots and Systems (2018), and Learning Curve (2019) emerged during the recent decade. Seventeen of twenty keywords (85%) attained peak frequencies during 2024–2025, reflecting marked acceleration in research productivity. The recent quinquennium (2021–2025) accounted for substantial proportions of total occurrences across dominant themes: Robotic Surgery (49.3%), Microsurgery (49.5%), Transoral Robotic Surgery (63.9%), and Reconstruction (68.8%), indicating intensified scholarly attention concentrated within the contemporary research landscape.

### Thematic evolution and conceptual progression

**Fig. 7:**
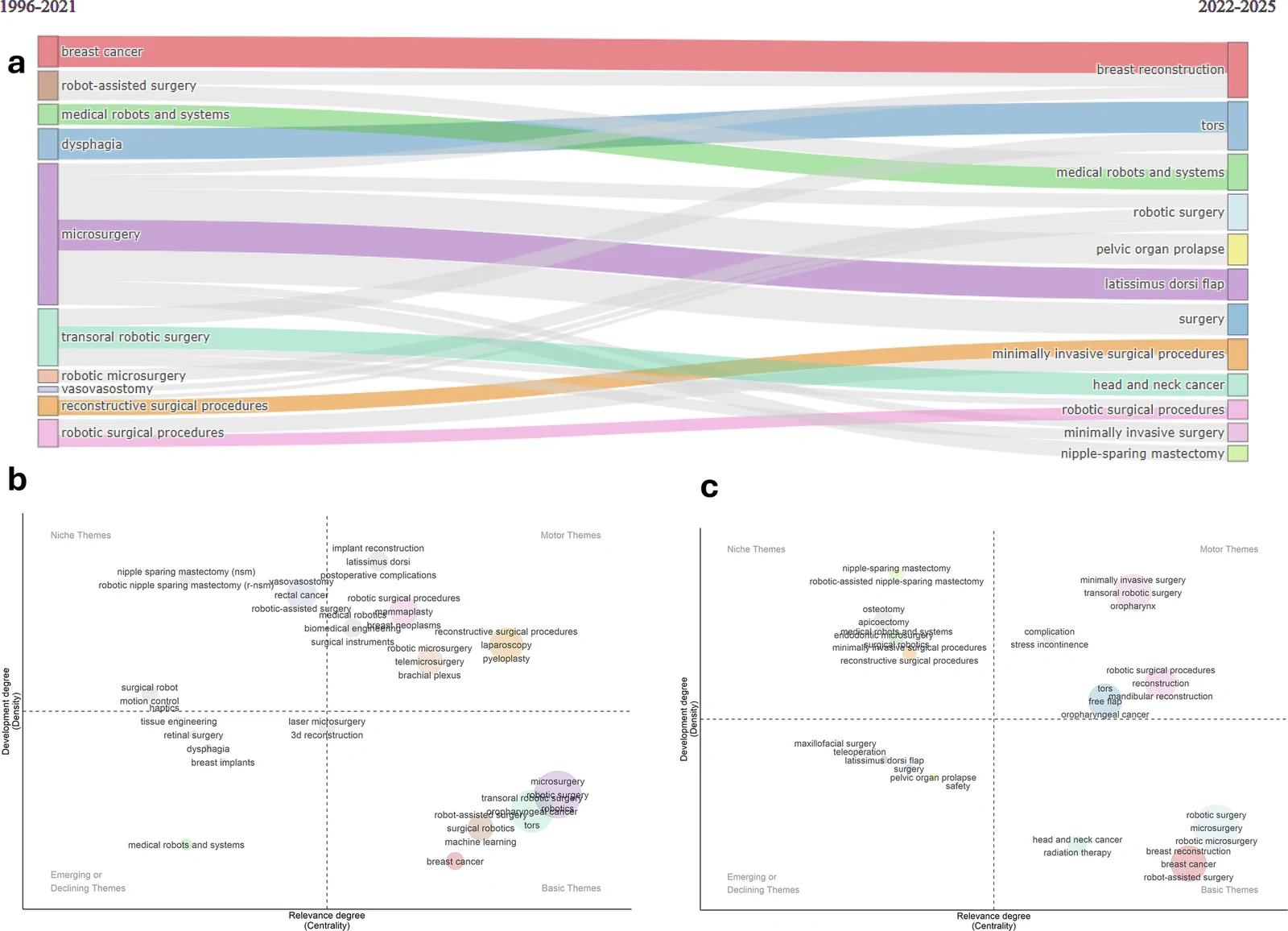
Thematic evolution and conceptual progression in robot-assisted plastic and reconstructive surgery research across two temporal phases. **a** illustrates a Sankey diagram depicting thematic evolution trajectories from foundational period (1996–2021) to contemporary period (2022–2025), with arrow thickness proportional to keyword co-occurrence frequency between source and destination themes. **b**, **c** present thematic maps for respective temporal phases, positioned according to Callon’s centrality (*x*-axis) and Callon’s density (*y*-axis). Four quadrants delineate motor themes, basic themes, niche themes, and emerging or declining themes. Node dimensions reflect keyword occurrence frequency, demonstrating progressive thematic consolidation and disciplinary maturation.

Thematic evolution analysis across two discrete temporal phases (1996–2021 and 2022–2025) revealed 23 distinct evolutionary pathways originating from ten foundational source themes (Fig. 7a). The first period (1996–2021) exhibited research concentration around microsurgery (*n* = 82 occurrences), transoral robotic surgery (*n* = 51), reconstructive surgical procedures (*n* = 12), and robot-assisted surgery (*n* = 11), which collectively constituted 88.6% of the evolutionary substrate. The second period (2022–2025) demonstrated thematic diversification into 13 distinct destination themes, with robotic surgery emerging as the principal convergence point, receiving contributions from four antecedent themes (microsurgery, reconstructive surgical procedures, robotic microsurgery, and vasovasostomy).

Microsurgery demonstrated the most extensive thematic fragmentation, bifurcating into eight derivative themes, including robotic surgery (*n* = 34), breast reconstruction (*n* = 13), minimally invasive surgery (*n* = 11), surgery (*n* = 9), transoral robotic surgery (TORS, *n* = 5), latissimus dorsi flap (*n* = 4), pelvic organ prolapse (*n* = 3), and nipple-sparing mastectomy (*n* = 3). Transoral robotic surgery exhibited quaternary divergence, evolving into minimally invasive surgery (*n* = 23), TORS continuation (*n* = 11), head and neck cancer (*n* = 9), and robotic surgical procedures (*n* = 8). Breast reconstruction received convergent inputs from three independent sources: microsurgery, robot-assisted surgery, and breast cancer themes, accumulating 26 combined occurrences.

Thematic continuity analysis identified medical robots and systems as exhibiting the highest stability index (0.50), indicating sustained conceptual persistence across temporal boundaries. Robot-assisted surgery demonstrated moderate stability (0.20) while transitioning toward medical robots and systems, accompanied by terminological evolution from “robot-assisted surgery” to “surgical robotics.” The breast cancer-to-breast reconstruction pathway exhibited a stability index of 0.11, reflecting progressive specialization from oncological diagnosis toward reconstructive intervention.

The first-period thematic map (Fig. 7b) positioned microsurgery, transoral robotic surgery, and robotic surgery as basic themes occupying high-centrality, low-density regions, indicating broad relevance with limited internal development. Breast cancer and machine learning emerged as basic themes demonstrating substantial centrality without cohesive clustering. Medical robots and systems occupied the emerging/declining quadrant, characterized by low centrality and density values. The second-period map (Fig. 7c) revealed spatial repositioning, with robotic surgery, microsurgery, and breast reconstruction consolidating as basic themes maintaining high centrality. Minimally invasive surgery and transoral robotic surgery transitioned to motor theme status, exhibiting elevated density and centrality coordinates. Nipple-sparing mastectomy and robot-assisted nipple-sparing mastectomy persisted as niche themes, demonstrating high density but peripheral centrality, while medical robots and systems, osteotomy, and endodontic microsurgery remained in transitional or declining positions with diminished network integration.

### Conceptual mapping

**Fig. 8:**
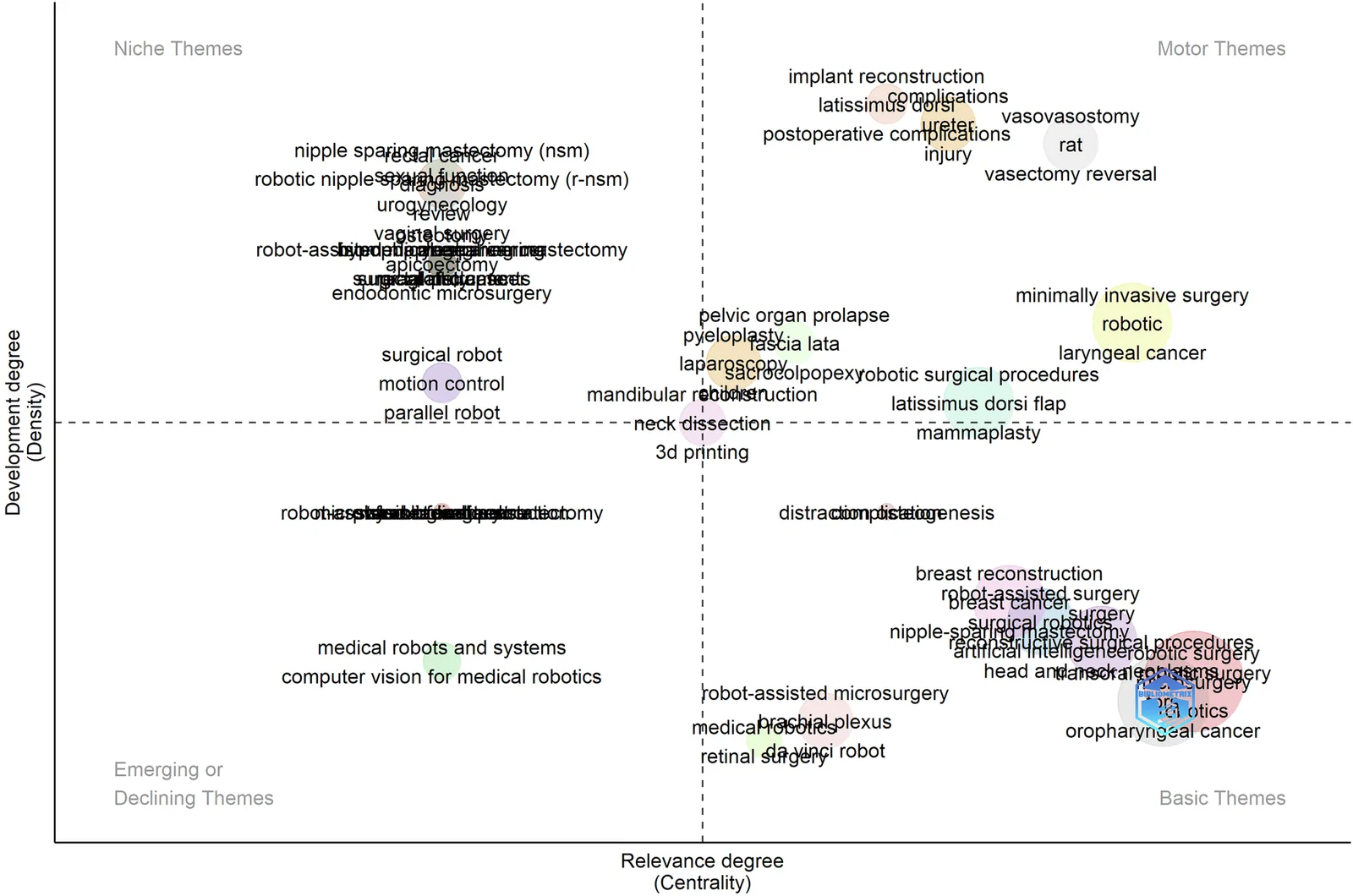
Thematic map of research clusters in robot-assisted plastic and reconstructive surgery based on author keywords. Clusters are positioned according to Callon’s centrality and density, delineating motor, basic, niche, and emerging or declining themes. Bubble size represents keyword occurrence frequency. Analysis performed using the Walktrap clustering algorithm via Bibliometrix software revealed 33 distinct thematic clusters across the research landscape.

Thematic analysis employing the Walktrap clustering algorithm identified 33 distinct research clusters from author keywords, mapped according to Callon’s centrality (relevance to research domain) and Callon’s density (internal development degree) (Fig. 8). Eight motor themes occupied the upper-right quadrant, representing well-developed and central topics including minimally invasive surgery (*n* = 92), robotic surgical procedures (*n* = 54), complications (*n* = 20), vasovasostomy (*n* = 20), and implant reconstruction (*n* = 8), collectively comprising 235 occurrences. Nine basic themes in the lower-right quadrant demonstrated high centrality but limited development, dominated by robotic surgery (*n* = 338), transoral robotic surgery (*n* = 185), and breast reconstruction (*n* = 60), totaling 710 occurrences. Nine niche themes exhibited specialized development with peripheral relevance, encompassing rectal cancer, robot-assisted nipple-sparing mastectomy, and osteotomy (56 occurrences). Seven emerging or declining themes occupied the lower-left quadrant, including medical robots and systems, force feedback, and physiological tremor (19 occurrences), indicating either nascent investigation or waning scholarly interest. This distribution reveals mature clinical applications dominating motor and basic quadrants, while technical innovations remain peripheral or underdeveloped. Thematic mapping based on core source dissemination was performed to triangulate author keyword-based findings (Fig. 8), confirming robotic surgery’s motor theme status through an independent analytical approach and validating thematic stability across methodological frameworks.

### Thematic mapping based on core source dissemination

**Fig. 9:**
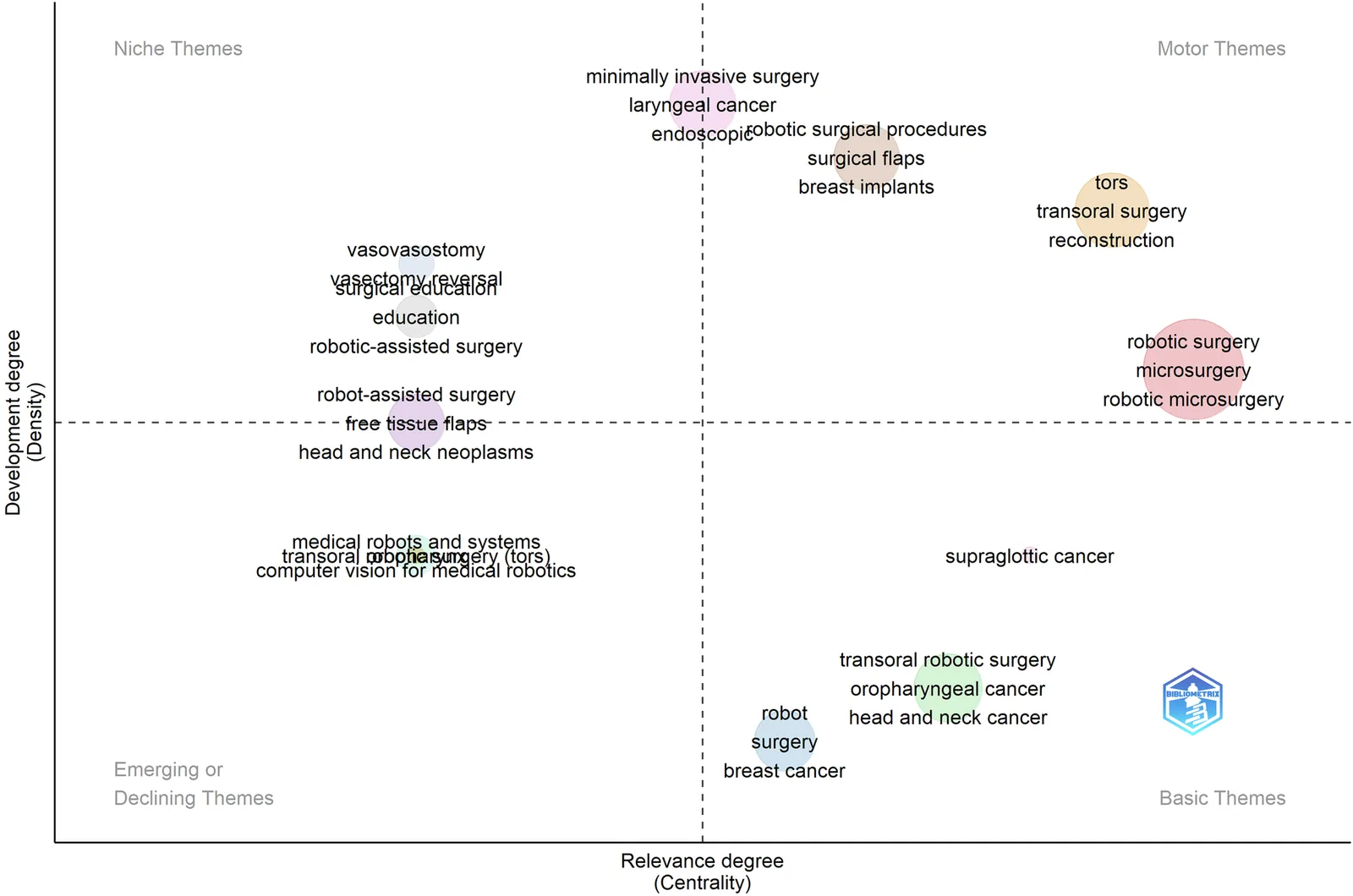
Thematic map of robot-assisted plastic and reconstructive surgery research based on Callon centrality and density. The map visualizes 13 thematic clusters distributed across motor, basic, niche, and emerging or declining quadrants, highlighting robotic surgery and microsurgery as dominant motor themes, with translational and disease-oriented clusters supporting a mature yet diversifying research landscape.

The thematic mapping identified 13 distinct clusters, reflecting the conceptual diversity and developmental heterogeneity of research in RAPRS. Among these, robotic surgery exhibits the highest Callon centrality (2.191) with substantial density (55.525), confirming its role as the primary motor theme driving the field (Fig. 9). Closely related clusters such as TORS (centrality = 0.905; density = 61.327), robotic surgical procedures (0.325; 63.690), and minimally invasive surgery (0.133; 80.816) demonstrate high internal development, highlighting mature and well-established research streams.

Clusters with moderate centrality, including transoral robotic surgery and supraglottic cancer, act as bridging themes between core robotic methodologies and disease-specific applications. In contrast, clusters with zero centrality—such as robot-assisted surgery, surgical education, medical robots and systems, vasovasostomy, and oropharynx—represent niche or emerging themes, characterized by specialization and limited connectivity to the broader knowledge structure. Collectively, the distribution of these 13 clusters indicates a mature research core centered on robotic and minimally invasive techniques, alongside peripheral themes that signal ongoing diversification and future innovation pathways.

### Trending topic analysis

**Fig. 10:**
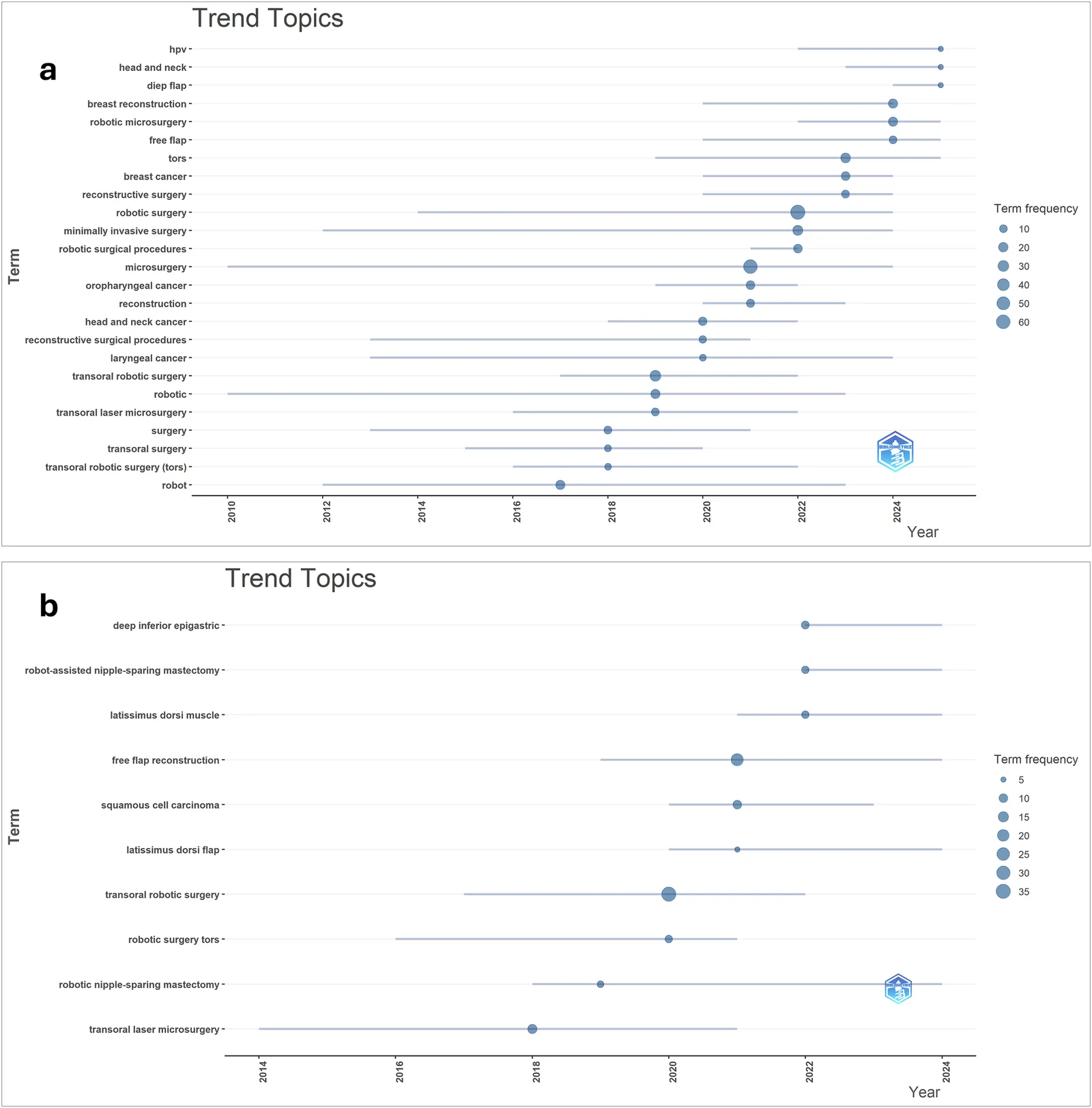
Temporal evolution of trending research topics in robot-assisted plastic and reconstructive surgery. **a** depicts the author keyword temporal dynamics, while **b** illustrates trigram term emergence patterns extracted from publication titles. Horizontal lines represent the temporal span of topic occurrence; circle positioning indicates the median year of emergence; circle diameter reflects term occurrence frequency.

Trending topic analysis employing Bibliometrix methodology revealed temporal evolution patterns of research themes derived from author keywords (Fig. 10a) and trigram terms extracted from publication titles (Fig. 10b). Visualization elements comprised horizontal lines indicating temporal emergence and persistence of each theme, with circle positioning denoting median year of occurrence and circle diameter representing term frequency magnitude.

Author keyword analysis (Fig. 10a) demonstrated the recent emergence of specialized reconstructive techniques, with deep inferior epigastric perforator flaps and robot-assisted nipple-sparing mastectomy appearing post-2022, while foundational terms including transoral robotic surgery, free flap reconstruction, and latissimus dorsi applications emerged during 2018–2020. Title-derived trigram analysis (Fig. 10b) revealed progressive thematic stratification, with early-period terms (2016–2018) encompassing generic robotic surgical terminology (“robot,” “transoral surgery”), mid-period expansion (2019–2021) incorporating oncological applications (“oropharyngeal cancer,” “head and neck cancer”), and contemporary concentration (2022–2024) emphasizing advanced microsurgical techniques (“robotic microsurgery,” “DIEP flap”) and oncoplastic breast reconstruction (“breast reconstruction,” “breast cancer”). The temporal progression collectively demonstrated disciplinary maturation from foundational robotic surgical paradigms toward specialized, anatomically specific reconstructive applications.

### CiteSpace co-citation, timeline, and cluster analysis (2021–2025)

**Fig. 11:**
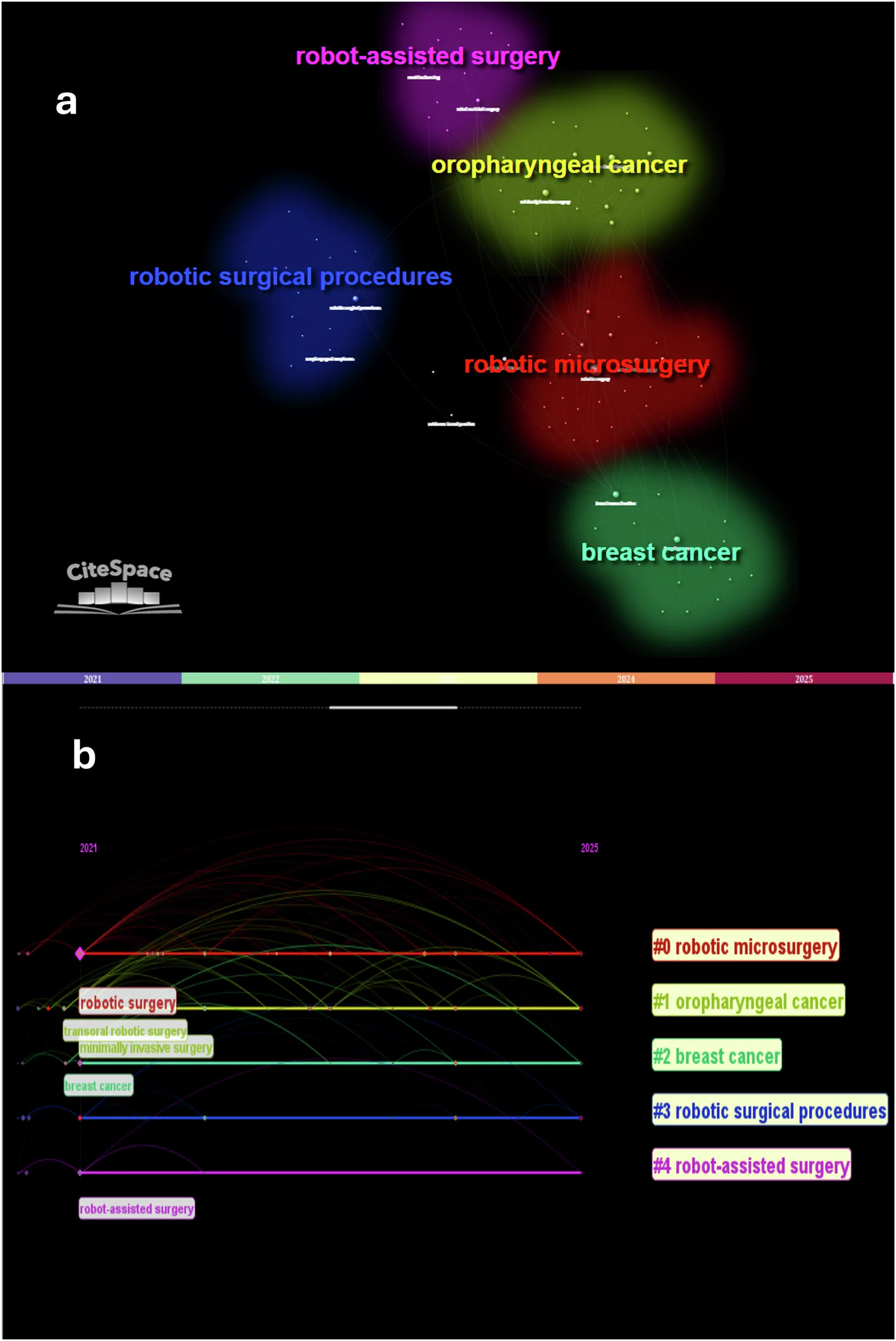
CiteSpace co-citation network, cluster map, and timeline visualization of robot-assisted plastic and reconstructive surgery research (2021–2025). **a** depicts the co-citation cluster structure highlighting six major thematic domains, with robotic microsurgery as the central intellectual hub linking oncologic and procedural applications. Panel **b** illustrates the temporal evolution of these clusters.

CiteSpace analysis revealed a well-structured and coherent knowledge network in RAPRS, comprising six major clusters with high silhouette values (0.879–0.996), indicating strong thematic consistency and reliable cluster separation (Fig. 11a). Robotic microsurgery (Cluster #0) emerged as the most structurally central cluster within the co-citation network, exhibiting the highest centrality (0.36) and degree (38), and spanning the entire study period (2021–2025). This cluster anchors the literature around microsurgical reconstruction, including free flap techniques and breast reconstruction, and functions as the primary connector linking diverse research themes.

Disease-oriented clusters, notably oropharyngeal cancer (Cluster #1) and breast cancer (Cluster #2), represent major clinical application domains where robotic platforms are translated into reconstructive oncology, particularly through transoral robotic surgery and robot-assisted nipple-sparing mastectomy. Robotic surgical procedures (Cluster #3) and robot-assisted surgery (Cluster #4) act as cross-cutting methodological clusters, bridging procedural innovation with disease-specific applications. The timeline visualization (Fig. 11b) demonstrates sustained and parallel thematic trajectories rather than abrupt citation bursts, indicating incremental consolidation and maturation of the field. Artificial intelligence–related nodes indicate a nascent research signal, suggesting early-stage exploration of AI-assisted robotic workflows rather than established thematic integration. Consistent with this interpretation, AI-related nodes exhibited low betweenness centrality and no detectable citation burst signals, indicating peripheral positioning within the RAPRS knowledge network. Overall, the findings identify microsurgical reconstruction as a structurally central theme within the bibliometric network, with expanding oncologic applications and gradual technological diversification across related research domains.

### Seminal studies analysis

**Fig. 12:**
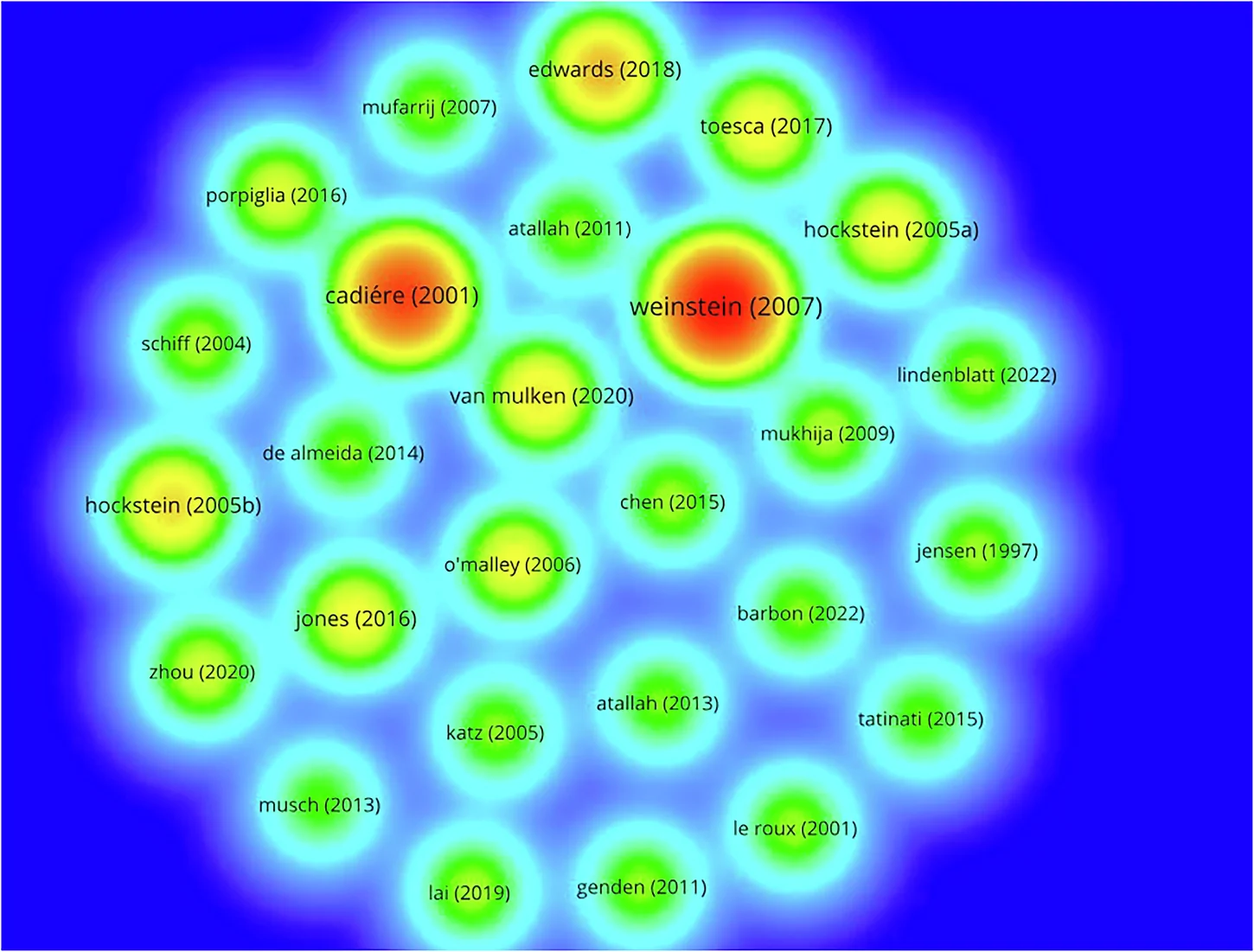
Density visualization of seminal highly cited documents in robot-assisted plastic and reconstructive surgery research. VOSviewer-generated citation density map encompasses 27 foundational studies exceeding a 70-citation threshold. Heat gradient intensity reflects local citation density concentration, with labeled nodes identifying lead authors and publication years.

Density visualization of highly cited foundational studies revealed thematic concentration across transoral robotic surgery, urological applications, and reconstructive microsurgery domains (Fig. 12). Weinstein (2007) commanded the highest citation frequency (550 citations), addressing robot-assisted pharyngeal and laryngeal microsurgery. Cadière (2001) contributed seminal work on robotic laparoscopic surgical feasibility (409 citations). Contemporary contributions included Edwards (2018) on intraocular robotic surgery (205 citations), Van Mulken (2020) on robotic supermicrosurgery for lymphedema management (154 citations), and Toesca (2017) on pioneering robotic nipple-sparing mastectomy (144 citations). Additional foundational studies encompassed Hockstein’s transoral robotic microlaryngeal investigations (2005a,b; 314 combined citations), O’Malley’s radical tonsillectomy protocols (2006; 130 citations), Porpiglia’s robot-assisted prostatectomy techniques (2016; 116 citations), and Jones’s methodological advances (2016; 135 citations).

## Discussion

This study provides the first comprehensive, data-driven synthesis of global research on robot-assisted plastic and reconstructive surgery (RAPRS) based exclusively on original research articles. The findings demonstrate a rapidly expanding and highly collaborative research landscape, with robotic microsurgery emerging as the central intellectual and clinical axis of the field. Consistent with the study hypothesis, the results reveal a mature knowledge structure anchored in reconstructive microsurgery, alongside accelerating clinical translation into oncologic reconstruction and early integration of advanced computational and artificial intelligence–enabled robotic technologies.

The exponential growth trajectory mirrors patterns observed in bibliometric analyses of other minimally invasive surgical technologies, including laparoscopic surgery^17,18^ and endovascular interventions^19,20^, where technological maturation precipitates scholarly proliferation. The temporal decoupling between publication volume and citation impact reflects a universal bibliometric phenomenon wherein foundational studies accumulate disproportionate citations through sustained reference in subsequent literature, while contemporary publications require extended observation periods to achieve comparable impact^21^. This pattern parallels findings reported in robotic surgery bibliometric investigations across urology and general surgery, where seminal proof-of-concept publications from the early 2000s commanded citation dominance despite representing a minority of corpus volume^22,23^.

The increase in publication output observed in recent years suggests growing research activity and clinical interest in RAPRS. However, given partial-year data for 2025 and citation-window effects, this trend should be interpreted as increasing scholarly momentum rather than definitive evidence of technological consolidation^24^. However, the citation concentration among early-period publications may also reflect methodological artifacts, as highly cited foundational studies establish terminological frameworks and reference standards that subsequent investigations perpetually cite, creating self-reinforcing citation networks. Longitudinal tracking of recent publications’ citation trajectories will determine whether contemporary contributions ultimately achieve comparable impact or whether citation consolidation among seminal works represents enduring structural characteristics of robotic surgical scholarship.

The geographic concentration mirrors patterns documented in bibliometric analyses of robotic cardiac surgery and urological robotics, where research productivity correlates strongly with national healthcare expenditure and surgical robot installation density^22,23^. USA dominance reflects substantial Da Vinci system market penetration, with approximately 70% of global installations concentrated in North American institutions^25^. China’s post-2015 acceleration parallels national strategic investments in surgical robotics manufacturing and clinical implementation programs, documented in analyses of Chinese surgical innovation trajectories^26^.

The disproportionate citation influence commanded by North American and European institutions, despite comparable Asian publication volumes, suggests systematic differences in journal impact factors, English-language publication advantages, and international research visibility. Previous bibliometric investigations across surgical subspecialties consistently demonstrate 2-3 year citation lag periods for non-English-speaking nations, potentially explaining South Korea’s robust productivity yet comparatively diminished citation accumulation.

European intra-regional collaboration density reflects established multinational research consortia and European Union funding mechanisms promoting cross-border partnerships, contrasting with Asia’s fragmented networks lacking comparable supranational research infrastructure^27^. The USA-France collaborative prominence replicates patterns observed in laparoscopic surgery bibliometrics, suggesting sustained institutional partnerships transcending individual technological platforms^28^. Africa and South America’s limited representation likely reflects multiple structural factors, including the high cost of robotic surgical systems, variations in subspecialty infrastructure, and barriers related to English-language publication and database indexing^29^. The observed international co-authorship rate of 20.45% appears modest in comparison with bibliometric patterns reported in other robotic and minimally invasive surgical fields, where collaboration networks are similarly shaped by a limited number of high-resource countries and institutionally concentrated research clusters.

The deviation from Lotka’s theoretical law, while suggesting limited sustained individual engagement, requires cautious interpretation, acknowledging structural factors shaping surgical research publication patterns. Robotic surgery represents a technological platform rather than a comprehensive surgical specialty, wherein investigators may contribute occasional empirical studies within broader careers spanning multiple techniques and approaches. Additionally, academic medical centers frequently generate robotic surgery research through rotating trainees (residents, fellows) supervised by core faculty, creating publication patterns reflecting institutional continuity despite individual trainee transition. Distinguishing between individual investigator commitment, institutional programmatic investment, and technology-driven collaboration models requires longitudinal cohort analyses beyond the scope of bibliometric methodology.

The most extensively cited foundational studies established proof-of-concept paradigms across multiple surgical subspecialties. Weinstein et al. pioneered transoral robotic surgery (TORS) radical tonsillectomy, achieving 93% negative margin resection in 27 patients with tonsillar squamous cell carcinoma, thereby establishing TORS as a viable oncological alternative to traditional open approaches^30^. Cadière et al. demonstrated robotic laparoscopic feasibility across 146 procedures spanning cholecystectomy, antireflux surgery, and tubal reanastomosis, validating the Da Vinci system’s clinical applicability and establishing foundational technical parameters for subsequent robotic surgical development^12^.

Contemporary contributions expanded robotic applications into supramicrosurgical domains. Van Mulken et al. reported the first-in-human robotic supermicrosurgery for lymphovenous anastomosis in breast cancer-related lymphedema, demonstrating dramatic operative time reduction (33 to 16 min) and technical superiority for 0.3-0.8 mm vessel anastomosis^7^. Edwards et al. established intraocular robotic surgery feasibility through randomized trials comparing robot-assisted versus manual epiretinal membrane dissection, demonstrating equivalent surgical success with statistically comparable retinal microtrauma rates^13^. Toesca et al. pioneered robotic nipple-sparing mastectomy in 29 breast cancer patients, achieving 93.1% technical success with minimal conversion rates (6.9%) and absence of major complications^31^. Hockstein et al. (2005a, 2005b) validated robotic microlaryngeal surgery through cadaveric dissections and airway mannequin feasibility studies, demonstrating precise tissue manipulation, tremor elimination, and endolaryngeal suturing capabilities^3,4^. These seminal investigations collectively established robotic surgery’s technical feasibility, safety profile, and clinical efficacy across reconstructive, oncological, and microsurgical domains.

Temporal stratification (Figs. 6 and 7) indicates an early phase characterized by foundational proof-of-concept investigations in microsurgery and robotic surgery (2001–2002), followed by progressive diversification into clinical applications across reconstructive and oncologic domains. The extensive fragmentation of microsurgery into multiple derivative themes parallels evolutionary patterns observed in laparoscopic surgery, in which single technological platforms enabled increasing procedural specialization^32^. In contrast, the emergence of robotic surgery as a principal convergence point indicates disciplinary consolidation around standardized robotic platforms, mirroring the transition observed in robotic urological surgery from heterogeneous techniques to unified surgical paradigms^33^. Thematic stability indices further revealed divergent evolutionary pathways, with medical robots and systems demonstrating sustained engineering-driven persistence, while the breast cancer–to–breast reconstruction progression reflected increasing clinical specialization and integration within multidisciplinary oncologic care^34^. Quadrant repositioning (Fig. 7) across temporal phases—particularly the advancement of minimally invasive surgery and transoral robotic surgery to motor theme status—signaled maturation from exploratory investigation to central, well-developed research domains. This thematic evolution reflects progressive diversification and consolidation within the research literature, consistent with patterns reported in bibliometric studies of emerging surgical technologies^35^.

The quadrant distribution (Fig. 8) reveals characteristic patterns of maturing surgical technologies, wherein clinical applications achieve motor theme status while foundational engineering innovations remain peripheralized, mirroring trajectories documented in robotic urological surgery bibliometrics where procedural refinements superseded technological development as research priorities^36,37^. The predominance of basic themes (robotic surgery, transoral robotic surgery, breast reconstruction) with high centrality but limited density indicates broad disciplinary relevance without cohesive theoretical frameworks, contrasting with laparoscopic surgery’s evolution, where established motor themes demonstrated both centrality and internal coherence. The relegation of force feedback and medical robots to emerging/declining quadrants suggests a disconnect between engineering research and clinical implementation, paralleling findings in surgical robotics reviews wherein haptic technology development progressed independently from clinical adoption trajectories. Bridging themes connecting core methodologies to disease-specific applications reflect multidisciplinary care model integration, consistent with oncoplastic surgery evolution documented across breast reconstruction bibliometrics^38,39^. The dual thematic mapping (Figs. 8 and 9) convergence—author keywords and source dissemination both identifying robotic surgery as a dominant motor theme—validates thematic stability across methodological approaches, enhancing confidence in the identified knowledge structure. Collectively, the distribution suggests increasing emphasis on clinical applications alongside continued technological development within the RAPRS research landscape.

The exceptional silhouette values and sustained parallel trajectories^40^ indicate disciplinary consolidation around coherent intellectual foundations, contrasting with fragmented citation networks documented in nascent surgical technologies, where methodological heterogeneity impedes thematic coherence. Robotic microsurgery’s centrality as intellectual backbone mirrors patterns observed in minimally invasive surgery bibliometrics, where core technical platforms anchor diverse clinical applications through shared methodological frameworks^41^. The bifurcation into disease-oriented and methodology-oriented clusters reflects classic translational research architecture, wherein procedural innovations enable multiple oncological applications—a pattern extensively documented across surgical oncology evolution studies^42^.

The absence of abrupt citation bursts, replaced by incremental consolidation trajectories, suggests field maturation beyond experimental phases characterized by paradigm shifts and revolutionary breakthroughs^42^. This evolutionary pattern parallels robotic urological surgery’s bibliometric progression, where sustained incremental refinements superseded disruptive innovations following initial technology validation^33,43^. Emerging artificial intelligence nodes signal nascent integration of computational technologies, consistent with broader surgical robotics trends documented in recent systematic reviews wherein machine learning applications transition from peripheral investigations toward mainstream research priorities. Collectively, the network architecture demonstrates a transition from technology-centric inquiry toward application-driven scholarship, characteristic of established surgical subspecialties documented in longitudinal innovation analyses^44^.

Based on the trend analysis (Fig. 10), deep inferior epigastric artery perforator (DIEP)–related robotics has clearly emerged as a prominent and rapidly expanding research theme within RAPRS^45^. Large multicenter consensus work has established robot-assisted microsurgery as a clinically viable and increasingly standardized platform, with lymphatic and flap-based procedures identified as key current and future indications^46^. Building on this foundation, fully integrated robotic workflows have been successfully applied to oncoplastic breast surgery, demonstrating the feasibility of combining robotic mastectomy with robotic DIEP flap harvest while achieving excellent oncologic and reconstructive outcomes^47^. Parallel anatomical and imaging-driven investigations have refined robotic DIEP flap planning, including updated classifications of perforator and branching patterns critical for safe robotic harvest^48^ and advanced three-dimensional mapping techniques that enhance perforator identification and candidate selection^49^. Clinical outcome studies further support this trend, showing reduced postoperative pain, narcotic use, and donor-site morbidity with robot-assisted DIEP reconstruction compared with conventional abdominal free flaps^50^. Early national and regional milestone reports, including the first robot-assisted DIEP reconstructions in the United Kingdom and Eastern Europe, underscore accelerating global adoption and technical feasibility^51,52^. Broader reconstructive literature increasingly positions robotic flap harvest as central to minimizing morbidity and improving patient quality of life^53^, while high-volume single-center experiences confirm the safety and scalability of robot-assisted microsurgical anastomosis in free flap surgery^54^. Collectively, these converging clinical, anatomical, and technological advances explain the bibliometric emergence of “deep inferior epigastric” as a dominant contemporary theme and signal its role as a cornerstone of next-generation robotic reconstructive surgery.

Artificial intelligence-related publications represent a nascent peripheral signal (2.5% of corpus) rather than an established research trajectory, suggesting early-stage exploration rather than widespread integration. The temporal concentration during 2022-2025 indicates emerging investigator interest, though current bibliometric evidence does not support characterization of AI as a dominant or central theme within RAPRS scholarship.

The bibliometric patterns documented herein yield specific actionable insights for multiple stakeholder constituencies. For surgical training programs, robotic microsurgery’s positioning as a dominant motor theme indicates curricular prioritization of supermicrosurgical competencies, particularly lymphovenous anastomosis and free flap harvest techniques, which should constitute core components of contemporary plastic surgery residency and fellowship education. The identification of 14 core journals contributing 33.3% of publications provides evidence-based guidance for institutional library collection development and trainee literature surveillance strategies.

For research administrators and funding agencies, substantial geographic disparities—with Africa, South America, and Southeast Asia representing <3% of cumulative citations—indicate strategic investment opportunities in underrepresented regions possessing surgical robotics infrastructure but limited research productivity. The fragmented collaboration network architecture, wherein 17 of 55 highly productive authors occupy isolated or peripheral positions, suggests institutional incentive structures should prioritize international consortium development, multicenter registry establishment, and collaborative grant mechanisms to enhance knowledge integration.

For technology developers and translational investigators, the peripheral positioning of force feedback, haptic technology, and medical robots engineering themes within thematic mapping indicates a disconnect between engineering innovation and clinical implementation research. Accelerated clinical translation requires deliberate bridging mechanisms connecting engineering advances with surgical outcome investigations, potentially through interdisciplinary funding initiatives and collaborative research infrastructure development.

Several methodological limitations warrant acknowledgment. Exclusive reliance on Scopus indexing may exclude publications indexed solely in Web of Science, PubMed/MEDLINE, IEEE Xplore, or regional databases, introducing potential indexing bias. Restriction to English-language articles may underrepresent regional scholarship. Additionally, excluding reviews, consensus statements, and clinical guidelines—while strengthening focus on original research—may have omitted influential expert perspectives shaping the field.

This study provides a systematic bibliometric mapping of RAPRS research, integrating multiple analytical platforms to delineate intellectual foundations, collaboration patterns, and thematic evolution across three decades of empirical scholarship. Five principal findings emerged: exponential publication growth (15.9% annually) concentrated post-2018; substantial geographic concentration in North America (30.41%) and Europe (38.09%) with marked disparities in research infrastructure; thematic evolution from foundational transoral applications toward specialized oncoplastic reconstruction and supermicrosurgery; fragmented collaboration networks despite technological capabilities enabling global cooperation; and pronounced author productivity skewness (78.8% single-publication contributors) indicating episodic rather than sustained engagement. The temporal acceleration and thematic diversification reflect technological maturation, expanding clinical adoption, and progressive integration of artificial intelligence methodologies. However, collaboration network fragmentation and geographic inequities suggest structural barriers constraining knowledge democratization. The transition from generic robotic surgical paradigms toward anatomically-specific reconstructive applications demonstrates disciplinary consolidation, while peripheral positioning of force feedback and haptic technologies indicates a disconnect between engineering innovation and clinical implementation research.

For research administrators, core journal identification (14 sources contributing 33.3% of publications) informs strategic collection priorities. Funding agencies should address geographic disparities through targeted infrastructure investment in underrepresented regions. Clinical educators must integrate emerging competency domains, including robotic microsurgery and oncoplastic techniques, into training curricula. Future investigations should employ comparative effectiveness methodologies examining patient-reported outcomes, altmetric analyses capturing societal impact beyond traditional citations, and qualitative content analysis elucidating substantive innovations underlying highly cited contributions.

The bibliometric evidence indicates increasing research consolidation, accelerating publication productivity, and growing thematic specialization within RAPRS, suggesting continued expansion of scholarly activity alongside emerging integration of computational approaches.

## Methods

### Data source and search strategy

A comprehensive bibliometric literature search was conducted using the Scopus database (Elsevier), selected for its extensive multidisciplinary coverage and established use in citation-based and science-mapping analyses. The search strategy was specifically designed to capture scholarly research on robot-assisted applications in plastic and reconstructive surgery (RAPRS), while minimizing interdisciplinary noise from other surgical specialties with high robotic utilization. Robotic surgery–related terms were combined with core plastic, reconstructive, and esthetic surgery descriptors and applied to article titles and abstracts to ensure both sensitivity and topical specificity. The full Boolean search strategy is provided in Supplementary File 1. In this context, PRISMA was used solely to document the identification, screening, eligibility, and inclusion stages of database record selection, while relevance assessment relied on predefined objective criteria rather than manual study-level appraisal typical of systematic reviews.

**Fig. 13:**
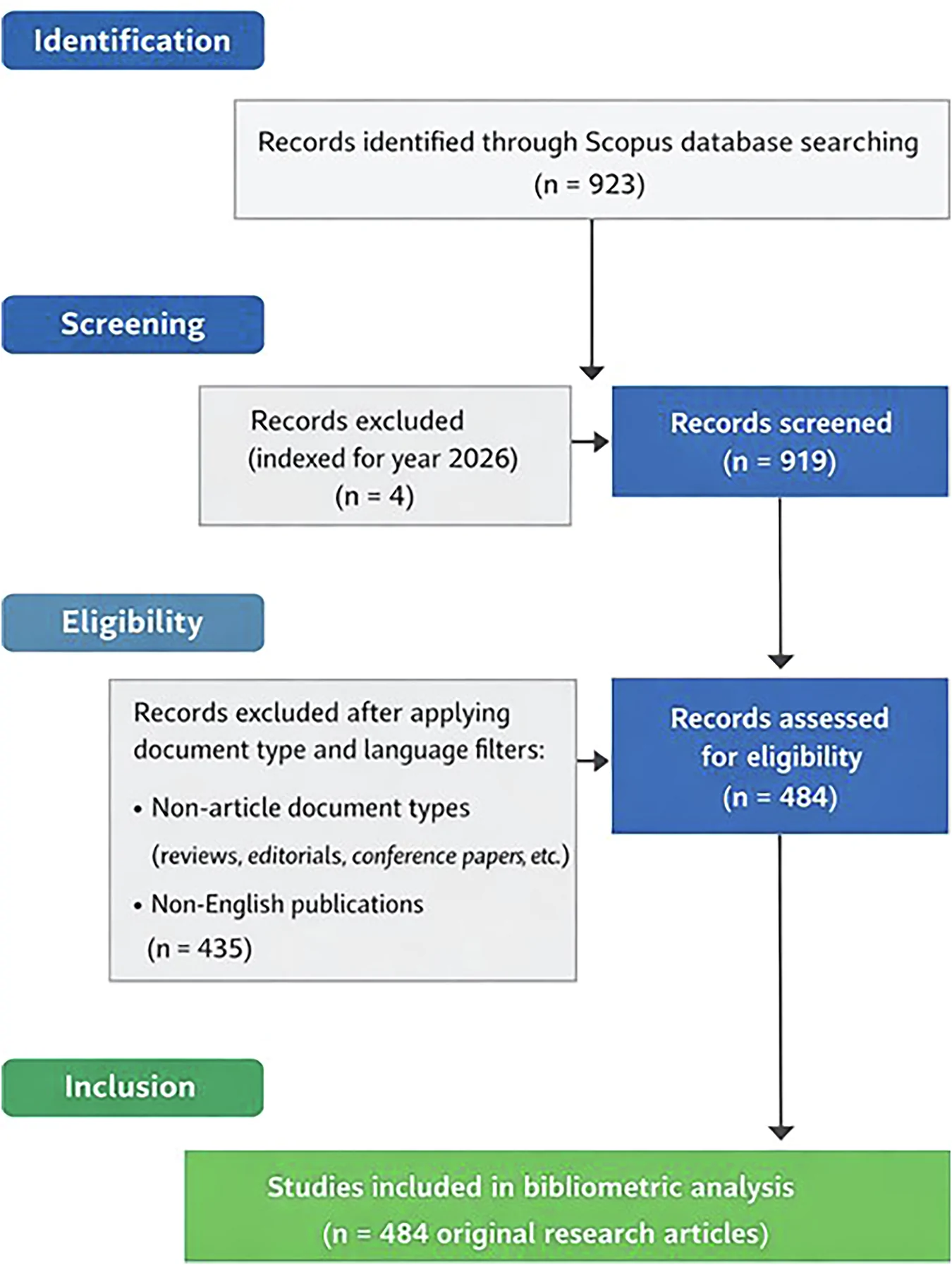
The study selection process followed the PRISMA (Preferred Reporting Items for Systematic Reviews and Meta-Analyses) guidelines, adapted for bibliometric research. During the identification phase, the Scopus search identified 923 records related to robot-assisted plastic and reconstructive surgery. In the screening phase, records indexed for the year 2026 (*n* = 4) were excluded, leaving 919 records that were subsequently filtered by document type and language. During the eligibility phase, the application of strict inclusion criteria—limited to original research articles and English-language publications—resulted in 484 eligible documents. In the inclusion phase, all 484 articles met the predefined inclusion criteria and were retained for the final bibliometric analysis. Inclusion criteria comprised publications addressing robot-assisted procedures in plastic, reconstructive, or esthetic surgery, original research articles, English-language publications, and records indexed in the Scopus database. Exclusion criteria included publications indexed for the year 2026, all non-article document types, and publications written in languages other than English.

Study selection followed the PRISMA guidelines adapted for bibliometric research (Fig. 13). The initial search yielded 923 records. To ensure dataset stability and reproducibility, records indexed for the year 2026 were excluded, leaving 919 documents for screening. Inclusion was restricted to English-language original research articles, while reviews, editorials, letters, conference papers, notes, short communications, and other non-primary document types were excluded. This data-driven approach emphasized empirical scientific contributions and reduced interpretive bias. Following refinement, a final dataset of 484 original research articles published between 1996 and 2025 was retained. No restrictions were applied regarding country, institution, or journal. Data for 2025 represent partial-year coverage (January–November) and are therefore not directly comparable with complete calendar years.

Bibliographic records were exported from Scopus in CSV format, including authorship data, institutional affiliations, abstracts, keywords, cited references, and citation metrics, which were used for performance analysis, network visualization, and thematic mapping. Broad reconstructive surgery terminology was intentionally retained to capture robotic microsurgical innovations that cross traditional specialty boundaries, recognizing that foundational robotic techniques developed in fields such as urology and otolaryngology have informed plastic surgical applications through shared microsurgical principles. Consequently, the dataset includes publications from plastic surgery, otolaryngology, urology, and robotics-oriented journals, reflecting the multidisciplinary dissemination of robotic reconstructive techniques.

### Search validation and data extraction

Literature search execution, data extraction, and bibliometric processing were performed using algorithmic database queries and established bibliometric software platforms (Bibliometrix, VOSviewer, and CiteSpace), minimizing subjective interpretation inherent in traditional systematic review screening. Search query validation and keyword consolidation were conducted independently by two investigators. To verify search sensitivity and inclusion accuracy, a random sample of retrieved records was manually screened for topical relevance, and representative benchmark studies in robotic microsurgery and DIEP flap reconstruction were cross-checked to confirm capture by the search query. By restricting inclusion to algorithmically retrieved original research articles meeting predefined objective criteria (document type, language, and indexing), this approach substantially reduced inter-rater variability and subjective bias compared with conventional systematic reviews reliant on manual relevance assessment.

### Bibliometric analysis and visualization tools

Bibliometric data analysis was conducted using a combination of Bibliometrix (version 4.1) implemented through the R statistical environment (version 4.3.1), VOSviewer (version 1.6.20), and CiteSpace (version 6.2.R4)^55–57^. Bibliometrix, developed in Italy, was used to perform descriptive bibliometric analysis, including annual publication trends, citation metrics, source impact analysis based on Bradford’s Law, thematic evolution analysis, trending topic analysis, conceptual structure mapping, and thematic mapping based on Callon’s centrality and density. Parameters applied included full counting, keyword unification through manual thesaurus harmonization, the Walktrap clustering algorithm for conceptual mapping, and a minimum keyword occurrence threshold of five^55^.

VOSviewer was employed for network-based visualizations, including international collaboration networks, author collaboration networks, author co-citation analysis, source co-citation analysis, and index keyword co-occurrence mapping. International collaboration networks were generated by applying a minimum threshold of five documents per country, resulting in 20 eligible countries for network construction. Author collaboration analysis was performed using a minimum threshold of five publications per author, yielding 55 authors included in the collaboration network. Author co-citation analysis was conducted by applying a minimum co-citation threshold of five citations per author, which identified 36 highly cited authors distributed across six clusters. Source co-citation analysis utilized a minimum threshold of five publications per source, resulting in 20 journals included in the co-citation network. Index keyword co-occurrence mapping was conducted using a minimum occurrence threshold of five keywords, producing a network of 430 keywords from the total corpus. All analyses were executed using the association strength normalization method, a full counting approach, and modularity-based clustering with layout optimization to ensure robust network structure and interpretability.

CiteSpace, developed in the USA, was utilized to examine the intellectual structure and temporal dynamics of the field through co-citation analysis, cluster analysis, and timeline visualization. Parameters included time slicing from 2021 to 2025 with one-year per slice, selection criteria based on g-index (*k* = 25), pruning using pathfinder and pruning sliced networks, and cluster labeling using log-likelihood ratio (LLR). Structural metrics such as betweenness centrality, silhouette values, citation burst detection, and sigma scores were calculated to assess cluster cohesion, thematic influence, and evolutionary patterns.

## Supplementary information

Supplementary file 1. Full search strategy

## Data Availability

The datasets generated and analyzed during the current study are not publicly available due to licensing restrictions associated with the Scopus database, but are available from the corresponding author on reasonable request.

## Code availability

No custom code was developed for this study. Bibliometric analyses were conducted using Bibliometrix (R version 4.3.1), VOSviewer (version 1.6.20), and CiteSpace (version 6.2.R4).
