# Supplementary material for: Research landscape of robot-assisted reconstructive and plastic surgery
Source: NPJ Digit Med. 2026 Jul 18;9:875. doi: 10.1038/s41746-026-03004-7 (PMC13748531; doi:10.1038/s41746-026-03004-7)
Supplement: Supplementary file 1 [file 41746_2026_3004_MOESM1_ESM.docx]

( ( TITLE ( "robotic-assisted" OR "robot assisted" OR "da Vinci" OR "robotic surgery" ) OR ABS ( "robotic-assisted" OR "robot assisted" OR "da Vinci" OR "robotic surgery" ) ) AND ( TITLE ( "plastic surgery" OR "reconstructive surgery" OR "aesthetic surgery" OR "breast reconstruction" OR "free flap" OR "microsurgery" ) OR ABS ( "plastic surgery" OR "reconstructive surgery" OR "aesthetic surgery" OR "breast reconstruction" OR "free flap" OR "microsurgery" ) ) )

923 document results

( ( TITLE ( "robotic-assisted" OR "robot assisted" OR "da Vinci" OR "robotic surgery" ) OR ABS ( "robotic-assisted" OR "robot assisted" OR "da Vinci" OR "robotic surgery" ) ) AND ( TITLE ( "plastic surgery" OR "reconstructive surgery" OR "aesthetic surgery" OR "breast reconstruction" OR "free flap" OR "microsurgery" ) OR ABS ( "plastic surgery" OR "reconstructive surgery" OR "aesthetic surgery" OR "breast reconstruction" OR "free flap" OR "microsurgery" ) ) ) AND ( EXCLUDE ( PUBYEAR , 2026 ) )

919 document results

( ( TITLE ( "robotic-assisted" OR "robot assisted" OR "da Vinci" OR "robotic surgery" ) OR ABS ( "robotic-assisted" OR "robot assisted" OR "da Vinci" OR "robotic surgery" ) ) AND ( TITLE ( "plastic surgery" OR "reconstructive surgery" OR "aesthetic surgery" OR "breast reconstruction" OR "free flap" OR "microsurgery" ) OR ABS ( "plastic surgery" OR "reconstructive surgery" OR "aesthetic surgery" OR "breast reconstruction" OR "free flap" OR "microsurgery" ) ) ) AND ( EXCLUDE ( PUBYEAR , 2026 ) ) AND ( LIMIT-TO ( DOCTYPE , "ar" ) ) AND ( LIMIT-TO ( LANGUAGE , "English" ) )

484 document results
